# EGFR-19del nuclear translocation increases HDAC7 expression inhibiting the Hippo pathway and exacerbating TKI resistance in lung adenocarcinoma

**DOI:** 10.7150/thno.128873

**Published:** 2026-04-08

**Authors:** Yuheng Feng, Minghao Wang, Yang Liu, Xinhao Wu, Xuyong Lin, Qiang Han, Xuezhu Rong

**Affiliations:** 1 Department of Pathology, First Hospital and College of Basic Medical Sciences of China Medical University, Shenyang, China.; 2 Department of Neurosurgery, First Hospital of China Medical University, Shenyang, China.; 3 Department of Pathology, First Hospital and College of Basic Medical Sciences of China Medical University, Shenyang, China.; 4 Department of Neurosurgery, First Hospital of China Medical University, Shenyang, China.; 5 Department of Pathology, First Hospital and College of Basic Medical Sciences of China Medical University, Shenyang, China.; 6 Department of Pathology, First Hospital and College of Basic Medical Sciences of China Medical University, Shenyang, China.; 7 Department of Pathology, First Hospital of China Medical University, Shenyang, China.

**Keywords:** EGFR, HDAC7, LATS1, YAP, Hippo pathway, drug resistance

## Abstract

**Rationale:**

Epidermal growth factor receptor (EGFR) “membrane-cytoplasmic-nuclear translocation” occurs in EGFR-19del lung adenocarcinoma (LUAD) following resistance to tyrosine kinase inhibitors (TKIs). This study aimed to elucidate the mechanism of TKI resistance conferred by nuclear EGFR-19del.

**Methods:**

RNA sequencing and immunohistochemistry were performed to assess histone deacetylase 7 (HDAC7) expression in LUAD with TKI resistance. Functional assays were performed both *in vitro* and *in vivo* to assess the effects of changes in HDAC7 expression on the malignant phenotype of LUAD cells and drug sensitivity to TKIs. Mass spectrometry and dual-luciferase assays were performed to verify the effect of changes in HDAC7 expression on the Hippo pathway. Chromatin immunoprecipitation and coimmunoprecipitation assays were conducted to clarify the potential role of EGFR-19del in the cell nucleus.

**Results:**

EGFR-19del nuclear translocation correlated with elevated HDAC7 expression in TKI-resistant cells. HDAC7 overexpression promoted malignancy and reduced TKI sensitivity, whereas HDAC7 knockdown or TSA treatment suppressed tumour growth and enhanced TKI sensitivity *in vivo*. Mechanistically, HDAC7 interacts with large tumour suppressor kinase 1 (LATS1) and promotes its deacetylation at K688, which reduced T1079 phosphorylation, thereby inhibiting the Hippo pathway. Concurrently, nuclear EGFR-19del acts as a coactivator to accelerate HDAC7 transcription through signal transducer and activator of transcription 3 (STAT3).

**Conclusions:**

We elucidated the underlying mechanism by which nuclear EGFR-19del inhibits the Hippo pathway; these results indicate that TKIs and HDAC inhibitors may serve as a potential therapeutic strategy to reduce drug resistance in LUAD with EGFR-19del.

## Introduction

Among malignant tumours, lung cancer has the highest incidence and mortality rate worldwide; in terms of pathological type, approximately 85% to 90% of cases are non-small cell lung cancer. Although surgical treatment offers the hope of disease-free survival for patients with early-stage lung cancer, the five-year survival rate for patients with advanced lung cancer is only approximately 15% 1. Recently, targeted therapeutic agents, especially tyrosine kinase inhibitors (TKIs, such as erlotinib, gefitinib, and osimertinib), have become the main targeted agents for lung cancer with EGFR somatic mutations, resulting in a significant increase in the survival of a subset of patients. However, after first-generation TKI drug treatment is administered, more than 50% of patients develop drug resistance 2. Although more than half of the cases of resistance to first-generation TKIs are due to the T790M mutation, the mechanism of resistance is still unclear in 30% of patients 3. Resistance can even occur after treatment with a third-generation TKI (osimertinib), and the mechanism of resistance is even more ambiguous than that with early-line TKIs 4. Therefore, an in-depth exploration of the mechanism of TKI resistance is not only beneficial for prognostic assessments and the development of treatment strategies for lung cancer patients but can also provide an experimental basis for the prevention of drug resistance and the development of new, effective targeted therapeutic agents.

The Hippo pathway signalling pathway that has been highly conserved throughout the evolution of species, and it is involved not only in regulating the volume and size of organs during development and influencing cellular development but also in its inactivation, which is closely related to the development of malignant tumours and drug resistance in humans 5,6. The molecular components of the pathway include upstream molecules such as FAT atypical cadherins (FATs), neurofibromin 2 (NF2; Merlin), WWC family proteins (WWCs), and angiomotin family proteins (AMOT proteins); a central kinase complex composed of mammalian sterile 20-like kinases 1/2 (MST1/2), Salvador family WW domain-containing protein 1 (SAV1), large tumour suppressor kinases 1/2 (LATS1/2), and MOB kinase activator 1 (MOB1); and downstream effector molecules, including Yes-associated protein (YAP) and transcriptional coactivator with a PDZ-binding motif (TAZ). When the Hippo pathway is activated, the central kinase complex undergoes a cascade phosphorylation reaction that promotes LATS1 phosphorylation (T1079), which in turn contributes to an increase in the level of p-YAP (S127). p-YAP can bind to 14-3-3 proteins in the cytosol and be degraded via the ubiquitin-proteasome pathway. In contrast, when Hippo pathway activity is inhibited, nonphosphorylated YAP enters the nucleus and binds to the transcription factor TEADs, which initiates the transcription of target genes, including connective tissue growth factor (*CTGF*), cysteine-rich angiogenic inducer 61 (*Cyr61*), and cyclin E (*CCNE*) 7-10. The epidermal growth factor receptor (EGFR) family plays important roles in tumorigenesis and development 11,12. Our group also found that mutant EGFR (19del) first undergoes membrane-plasma translocation after long-term treatment with TKIs and that the translocation of EGFR into the plasma inhibits the phosphorylation of MST-LATS by binding to salt-inducible kinase 2 (SIK2), leading to decreased YAP phosphorylation and accumulation in the nucleus; moreover, YAP entry into the nucleus initiates the transcription of *CTGF*, *CCNE*, etc., causing drug resistance 13. On the other hand, YAP can bind to EGFR in the plasma, and when YAP enters the nucleus, it also transports EGFR into the nucleus 13. Wild-type EGFR proteins entering the nucleus can act as transcriptional coactivators to promote the transcriptional activation of downstream oncogenes and exacerbate the emergence of TKI resistance 14. However, whether the translocation of EGFR-19del mutant proteins into the nucleus affects Hippo pathway activity and TKI resistance is unknown; notably, the relationship between nuclear EGFR-19del mutant proteins and the TKI response and the underlying mechanism have attracted our interest.

Histone deacetylase 7 (HDAC7) is a member of the histone deacetylase family. The gene is located on human chromosome 12 and has a molecular weight of approximately 100 Kda 15. The main functional domains of this protein include the myocyte enhancer factor 2 (MEF2) domain, the nuclear localization signal (NLS), the deacetylation (DAC) domain, and the nuclear export signal (NES) 15. To date, studies on the relationship between HDAC7 and tumours can be summarized as follows: high expression of HDAC7 in LUAD promotes lung carcinogenesis by inhibiting STAT3 activation 16. Furthermore, high HDAC7 mRNA levels are associated with poor prognosis for lung cancer patients 17. HDAC7 is overexpressed in choroidal melanoma (CM) tissues and promotes CM cell proliferation and metastasis through the c-Myc signalling pathway. Both of these effects can be inhibited when HDAC7 is inhibited by drug treatment 18. Studies of gliomas have shown that HDAC7 expression is upregulated in gliomas and promotes tumour cell proliferation and invasion. It increases Wnt signalling activity by deacetylating β-catenin, which in turn promotes the malignant progression of gliomas 19. Taken together, these findings suggest that HDAC7 has complex biological functions in different cancer contexts. Therefore, future studies are needed to more precisely define the role of HDAC7 in various cancers and to explore the possibility of targeting HDAC7 therapeutically.

In the present study, we propose that EGFR-19del in the nucleus can bind to the transcription factor STAT3 to promote the transcription of HDAC7, which binds to LATS1 and inhibits its phosphorylation by decreasing its acetylation, ultimately leading to the inhibition of Hippo pathway activity and the development of resistance to TKIs. The results of this study not only help to identify potential new molecular markers contributing to lung adenocarcinoma resistance to TKIs but also provide an important experimental basis for the application of new therapeutic strategies for the prevention and treatment of EGFR-19del-mutant lung adenocarcinomas in which resistance to TKIs occurs.

## Methods

### Culture of non-small cell lung cancer cell lines and induction of TKI resistance

Cell line culture and induction of TKI resistance: The lung adenocarcinoma cell line HCC827 (EGFR 19del, item no. CL-0094) used in this study was purchased from Prosperity Bio (China). Cells were identified by short tandem repeat (STR) sequencing and mycoplasma detection (negative results) and cultured in RPMI-1640 medium supplemented with 10% foetal bovine serum (Invitrogen, USA) in a constant-temperature incubator with 5% CO_2_ at 37 °C. The method for inducing TKI resistance and detecting the IC_50_ in cells was described in detail in a previous study 13.

### Cell transfection

The cells were transiently transfected using Lipofectamine 3,000 (no. L3000015; Invitrogen, USA) and Opti-MEM (no. 31985070; Invitrogen, USA) according to the manufacturer's instructions. Puromycin-containing medium (50 μg/mL Bioscience Co., Ltd.) was used for stable cell line selection. The information concerning the plasmids, shRNAs and siRNAs used in this study is listed in the Supplementary Materials and Methods.

### RNA-sequencing analysis

HCC827, HCC827-GR, HCC827-ER, and HCC827-OR cell line pellets were collected by an appropriate volume of TRIzol (no. 15596026CN; Thermo Fisher Scientific, USA) reagent to ensure the formation of a homogeneous lysate. After labelling and cryopreservation, the TRIzol-treated samples were quickly transported to Wuhan Kangce Technology Co., Ltd. (China) for RNA extraction and specific transcriptome sequencing. After the sequencing results were obtained, the differentially expressed genes were first analysed by comparing the HCC827-GR, HCC827-ER, and HCC827-OR cell lines with the HCC827 parental cells and filtering the list of significantly differentially expressed genes in each group using the same stringent criteria (e.g., |log2FC| > 1 and FDR < 0.05). The EVenn online tool (https://www.bic.ac.cn/EVenn/) was subsequently used to select the 4-group mode; the four differentially expressed gene list files were uploaded or pasted into Set A, Set B (GR), Set C (ER), and Set D (OR), and the names were determined to generate Venn diagrams to visualize the overlap and differences among the three drug-resistant sublines. The overlap and specificity relationships between the sets of differentially expressed genes were visualized.

### Pathology specimen collection and immunohistochemical staining

This study was approved by the Ethics Committee of China Medical University (no. AF-SOP-07-1.1-01) and followed the Declaration of Helsinki. Sixty paraffin-embedded lung adenocarcinoma specimens without preoperative radiotherapy were included (source: archived in the Department of Pathology of the First Affiliated Hospital of China Medical University, all of which were verified as having a deletion of the 19 locus of the EGFR gene by next-generation sequencing). Immunohistochemical staining was performed using the S-P method. The primary antibodies used were a mouse-derived polyclonal anti-HDAC7 antibody (1:50; sc-74563; Santa, Bolivia) and a rabbit-derived anti-mutant EGFR antibody (1:50; #4267; Cell Signaling Technology Danvers (CST), USA). PBS was used as a negative control. Five fields of view (400×) were randomly selected for each section, and 100 cells were counted in each field. Positive determination was graded on the basis of the percentage of positive cells as follows: 0 points (0%), 1 point (1-25%), 2 points (26-50%), 3 points (51-75%), and 4 points (> 76%). The staining intensity scores were as follows: 0 (no staining), 1 (light yellow granules), and 2 (dark yellow/yellowish-brown granules). A combined score, percentage score × intensity score, ≥ 4 was considered to indicate positive expression, and a score < 4 indicated negative expression.

### RNA extraction and RT‒qPCR

Assays were performed as described previously 13. The relative transcript levels of genes were normalized to the *GAPDH* mRNA level, and the sequences of the primers used for amplification are shown in Table 1.

### Protein extraction and immunoblotting

Protein extraction and immunoblotting assays were described in detail previously 13. Nucleoplasmic protein separation was performed according to the instructions provided in the Nucleoplasmic Separation Kit (P0028; Beyotime Biotechnology). Antibody information is described in the Supplementary Materials and Methods.

### Colony formation and Transwell assays

48 h after transfection, the surviving tumour cells were collected for colony formation and Transwell assays. The assays were described in detail in our previous study 13. The experiments were independently repeated in triplicate.

### EdU cell proliferation assay

Cells in the logarithmic growth phase were seeded into 6-well plates. When the confluence reached 60%, medium containing 10 μM EdU was added, and the cells were further cultured for 6 h at 37 °C under 5% CO_2_. The EdU detection kit (E-CK-A376; Elabscience, China) was subsequently used, and the cells were observed under a fluorescence microscope (Olympus, Japan).

### Subcutaneous tumour formation in nude mice

The animal experimental protocol was approved by the Ethics Committee of China Medical University (Approval No.: AF-SOP-07-1.1-01). Female BALB/c nude mice aged 4-6 weeks (SPF grade; Beijing HFK Biological Co., Ltd., China) were used to establish xenograft tumour models. Animals were randomly assigned to different treatment groups (n = 5) using a completely randomized grouping method. On day 0, 5 × 10⁶ tumour cells were inoculated subcutaneously into the right axilla of the mice. Starting 7 days post-inoculation, the tumour major diameter (L) and minor diameter (W) were measured twice weekly in a blinded manner, and the tumour volume was calculated using the formula TV = L × W^2^/2. Starting from week 3, the animals received either osimertinib (AZD-9291, 10 mg/kg, oral) or thiamphenicol A (TSA, 0.5 mg/kg, intraperitoneal injection). After the 8-week experiment concluded, all the mice were euthanized, and the xenograft tumours were completely resected and weighed.

### Immunofluorescence assay

Cells were seeded into 24-well plates and cultured to 50-60% confluence. The cells were fixed with 4% paraformaldehyde for 15-30 min and then blocked with 3% bovine serum albumin at room temperature for 1.5 h. The samples were incubated sequentially with primary antibody (1:50, overnight at 4 °C) and secondary antibody (1:200, 2 h at room temperature in the dark). Finally, the sections were mounted with DAPI-containing anti-fade mounting medium (P0131; Beyotime Biotechnology Co., Ltd., China) and imaged using confocal microscopy.

### Coimmunoprecipitation and mass spectrometry

The immunoprecipitation assay was described in detail previously 13. To control for nonspecific binding, IgG was used as a negative control. Proteins enriched by immunoprecipitation using either the HDAC7 antibody or the IgG control antibody were separated by SDS‒PAGE. Gel slices containing proteins that bound to overexpressed HDAC7 were analysed by nanoflow liquid chromatography‒tandem mass spectrometry (nanoLC‒MS/MS) using a Q Exactive mass spectrometer and an Easy nLC system from Thermo Fisher Scientific. The raw data were converted to MGF files with Proteome Discoverer 1.4. Subsequent peptide identification was performed in the UniProt database using Mascot software (v2.3.01), and the false discovery rate was controlled by a decoy database search (FDR < 0.05).

### Dual luciferase reporter gene assay

The assays were performed according to the instructions of the Dual Luciferase Reporter Gene Kit (E1910; Promega, Beijing, China) and were described in detail previously 13. The experiments were repeated in triplicate, and the results were standardized to those of the empty vector group and are reported as the means ± SDs.

### Chromatin immunoprecipitation (ChIP) and agarose gel electrophoresis

ChIP experiments were performed in STAT3 or Myc-EGFR(19del)-NLS-overexpressing HCC827-OR cells. Specifically, cells were cultured in 10 cm dishes to approximately 80% confluence and then cross-linked with 1% formaldehyde at room temperature for 10 min. ChIP experiments were subsequently performed using a commercial kit (Sigma‒Aldrich, St. Louis, MO, USA) with anti-STAT3 or anti-Myc-tag antibodies according to the manufacturer's protocol. Immunoprecipitated DNA was purified and analysed via PCR amplification and agarose gel electrophoresis.

### GST pull-down assay

The GST pull-down assay employed an *E. coli* system expressing the GST-LATS1 fusion protein. The strain was cultured in LB medium until the OD_600_ was 1.0-1.5 and then induced with 1 mM IPTG at 30 °C for 2 h. The cell pellet was resuspended after centrifugation and sonicated, and the supernatant was collected by centrifugation. This supernatant was incubated with the GST beads at 4 °C for 1 h. The beads were thoroughly washed with TBS until the OD_280_ was less than 0.05. Finally, the protein was eluted with SDS‒PAGE loading buffer for immunoblotting.

### Statistical analysis

All the data were statistically analysed using SPSS version 27.0. The chi-square test was used to examine the associations between HDAC7 expression and the EGFR-19del subcellular location status. An online Kaplan‒Meier analysis (https://www.kmplot.com) was performed to assess the relationship between HDAC7 expression and the overall prognosis of LUAD. Differences between groups were analysed using *t* tests or two-way analysis of variance (ANOVA). *P* values less than 0.05 were considered to indicate statistical significance.

## Results

### HDAC7 is highly expressed in TKI-resistant cells and tissues

**Figure 1:**
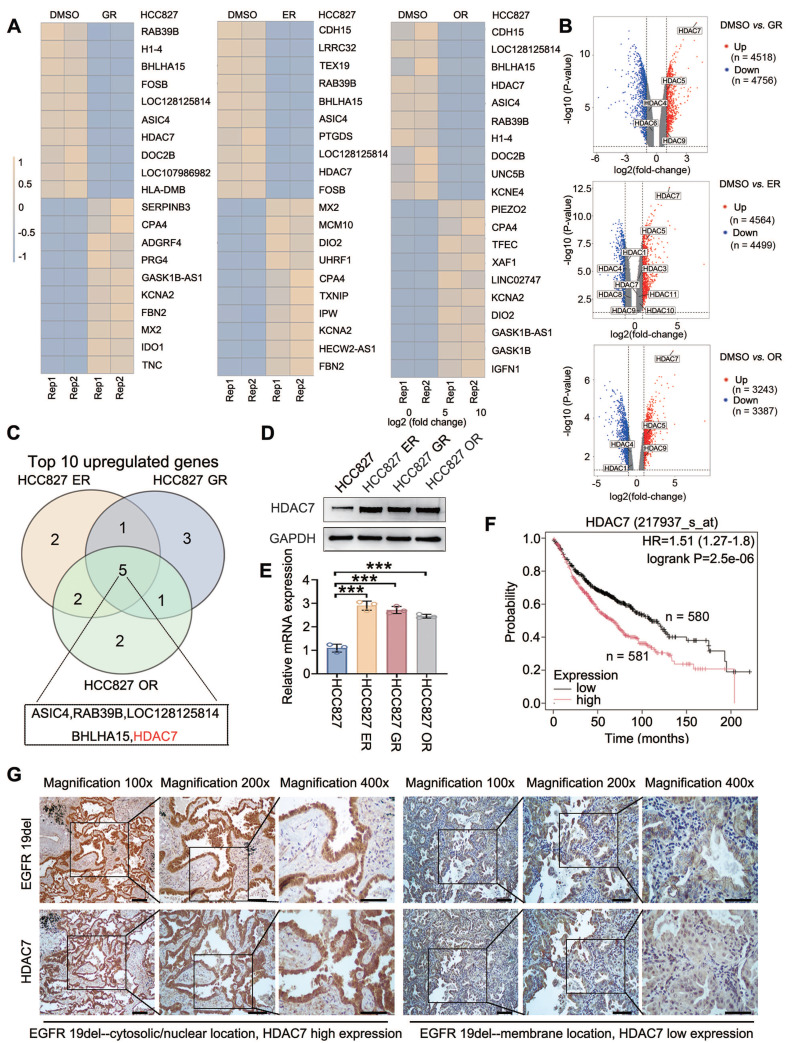
** HDAC7 expression is upregulated in TKI-resistant tissues and cells.** (A) RNA-seq analysis of parental HCC827 cells and gefitinib-resistant (HCC827-GR), erlotinib-resistant (HCC827-ER), and osimertinib-resistant (HCC827-OR) cell lines. The heatmaps show the top 10 genes whose expression was most significantly upregulated in each resistant cell line compared with that in parental HCC827 cells. (B) Volcano plots depicting genes that are differentially expressed between parental HCC827 cells and HCC827-GR, HCC827-ER, or HCC827-OR cells, as determined by RNA-seq analysis. The x-axis represents the log2-fold change, and the y-axis represents the -log10(P value). Upregulated and downregulated genes are shown in red and blue, respectively. The selected HDAC family members are annotated, with HDAC7 highlighted as one of the most significantly upregulated genes in all three resistant cell lines. (C) Venn diagram showing the overlap of the top 10 upregulated genes among HCC827-ER, HCC827-GR, and HCC827-OR cells, identifying common candidate genes associated with EGFR-TKI resistance. (D) Immunoblot showing the levels of HDAC7 protein expression in three TKI (gefitinib, erlotinib, and osimertinib)-resistant cell lines and the corresponding parental cell line; GAPDH was used as the internal reference protein. (E) RT‒qPCR detection of HDAC7 mRNA expression in cell lines resistant to the three TKIs and the parental cell line (*P* < 0.05). (F) The Kaplan‒Meier database was used to analyse the relationship between the HDAC7 expression level and patient overall survival (OS). (G) Immunohistochemical staining showing the localization of the EGFR-19del mutation in relation to HDAC7 positivity. Scale bar: 100 μm for 100× magnification; 50 μm for 200× magnification; 25 μm for 400× magnification.

HCC827 lung adenocarcinoma cell lines were subjected to sustained drug treatment with gefitinib, erlotinib and osimertinib, and the corresponding drug-resistant cell lines were named HCC827-GR (HCC827-gefitinib-resistant), HCC827-ER (HCC827-erlotinib-resistant), and HCC827-OR (HCC827-osimertinib-resistant) 13. By performing a comparative transcriptome sequencing (RNA-seq) analysis, we identified the top 10 genes whose expression was significantly upregulated in all three drug-resistant cell lines compared with that in the nonresistant cell line (HCC827) (HCC827-ER: log2FC: 4.102, *P* < 0.001; HCC827-GR: log2FC: 4.008, *P* < 0.001; HCC827-OR: log2FC: 4.775. *P* < 0.001) (**Figure 1A-C**). These results were verified by Western blot (**Figure 1D**) and RT‒qPCR (**Figure 1E**). Furthermore, Kaplan‒Meier online analysis (https://www.kmplot.com/) suggested that high HDAC7 expression was significantly associated with poor prognosis for lung adenocarcinoma patients (*P* < 0.001; **Figure 1F**). The data from our previous study suggest that the localization of mutant EGFR in the plasma/nucleus is strongly associated with shorter progression-free survival (PFS) and the occurrence of TKI resistance 13. Therefore, we further selected 60 lung adenocarcinoma pathological tissue samples with a deletion of the EGFR 19 locus and assessed the correlation between the different subcellular localizations of mutant EGFR-19del and the expression level of HDAC7 using immunohistochemistry. The results revealed that the percentage of HDAC7-positive cells in which mutant EGFR-19del was localized to the cell membrane (25/42, 59.5%) was significantly lower than that the percentage of HDAC7-positive cells in which EGFR-19del was localized to the plasma/nucleus (16/18, 88.9%) (**Figure 1G**). The difference was statistically significant (**Table 2**, *P* = 0.034). These experimental results indicate that HDAC7 is highly expressed in TKI-resistant lung adenocarcinomas and is particularly highly expressed in samples expressing mutant EGFR with plasma/nuclear translocation.

### HDAC7 promotes a malignant phenotype and attenuates drug sensitivity in TKI-resistant cells

**Figure 2:**
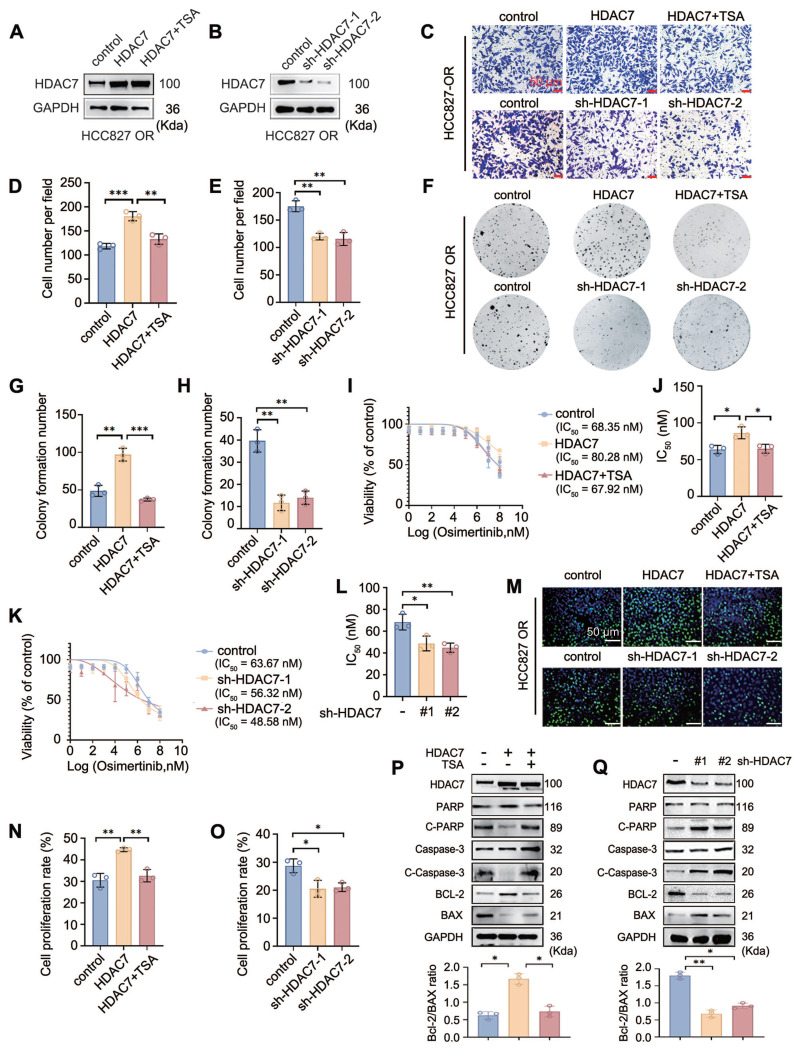
** HDAC7 promotes a malignant phenotype and reduces the sensitivity of HCC827-OR cells to TKIs.** (A, B) Western blot analysis of HDAC7 overexpression and knockdown efficiency in HCC827-OR cells. (C-E) Stromal gel invasion assay. The overexpression of HDAC7 increased invasion, and TSA reversed this change; knockdown of HDAC7 inhibited invasion (magnification: 200×). (F-H) Results of the cell colony formation assay. HDAC7 overexpression enhanced colony formation, and TSA reversed this effect; HDAC7 knockdown inhibited colony formation. (I-L) Drug sensitivity analysis (IC_50_ assay). HDAC7 overexpression increased the half maximal inhibitory concentration (IC_50_) of osimertinib, and TSA reversed this effect; HDAC7 knockdown decreased the IC_50_. (M-O) Results of the EdU cell proliferation assay. HDAC7 overexpression increased cell proliferation, and TSA reversed this effect; HDAC7 knockdown decreased proliferation. (P, Q) Western blot analysis of the bidirectional regulation of the expression of apoptosis-related proteins (cleaved caspase-3, cleaved PARP, Bcl-2, and Bax) after treatment with HDAC7. A histogram of the Bcl-2/Bax ratio was used to depict cell apoptosis. The data are presented as the means of three independent experiments. The columns represent the mean values, and the bars represent the standard deviations (SDs). *P < 0.05; **P < 0.01; and ***P < 0.001.

To further explore the functional role of HDAC7 in resistance to EGFR-TKIs, we selected the HCC827-ER and HCC827-OR cell lines and achieved stable overexpression and knockdown of HDAC7 by transfecting them with a Flag-HDAC7 plasmid carrying a lentiviral envelope and shRNA-HDAC7, respectively. The biological role of HDAC7 was verified using the broad-spectrum HDAC inhibitor troglitazone TSA (**Figure 2A-B, Figure S1A-B**). By performing a stromal gel invasion assay and a colony formation assay, we first revealed that the overexpression of HDAC7 significantly promoted HCC827-OR and HCC827-ER cell invasion (**Figure 2C-E, Figure S1C-E**) and colony formation (**Figure 2F-H, Figure S1F-H**) and that TSA administration and HDAC7 knockdown suppressed the proliferation and invasion of drug-resistant cells. We next determined the half maximal inhibitory concentration (IC_50_) of TKIs in drug-resistant cells after the bidirectional modulation of HDAC7 using a CCK8 assay to further verify the effect of HDAC7 on drug sensitivity, and the results revealed that the overexpression of HDAC7 significantly increased the IC_50_ values of TKIs in HCC827-OR and HCC827-ER cells; i.e., the overexpression of HDAC7 aggravated drug resistance, whereas TSA administration or HDAC7 knockdown significantly increased the sensitivity of drug-resistant cells to TKIs (**Figure 2I-L, Figure S1I-L**).

EdU proliferation assays were performed to assess the effect of HDAC7 on the proliferative capacity of drug-resistant cells, and the results similarly suggested that HDAC7 overexpression significantly promoted the proliferation of drug-resistant cells and that TSA treatment and HDAC7 knockdown reversed this pro-carcinogenic effect of HDAC7 (**Figure 2M-O, Figure S1M-O**). Finally, the effects of HDAC7 on the levels of apoptosis-related proteins were detected by Western blot; these experiments revealed that the overexpression of HDAC7 decreased the levels of Cleaved Caspase-3 (C-caspase3), Cleaved Poly (ADP-ribose) Polymerase (C-PARP), BCL-2-Associated X Protein (BAX), and B-cell lymphoma 2 (Bcl-2), but these changes were reversed by TSA treatment; conversely, the knockdown of HDAC7 upregulated the expression of the abovementioned apoptotic proteins (**Figure 2P-Q, Figure S1P-Q**).

### HDAC7 suppresses the Hippo signalling pathway

**Figure 3:**
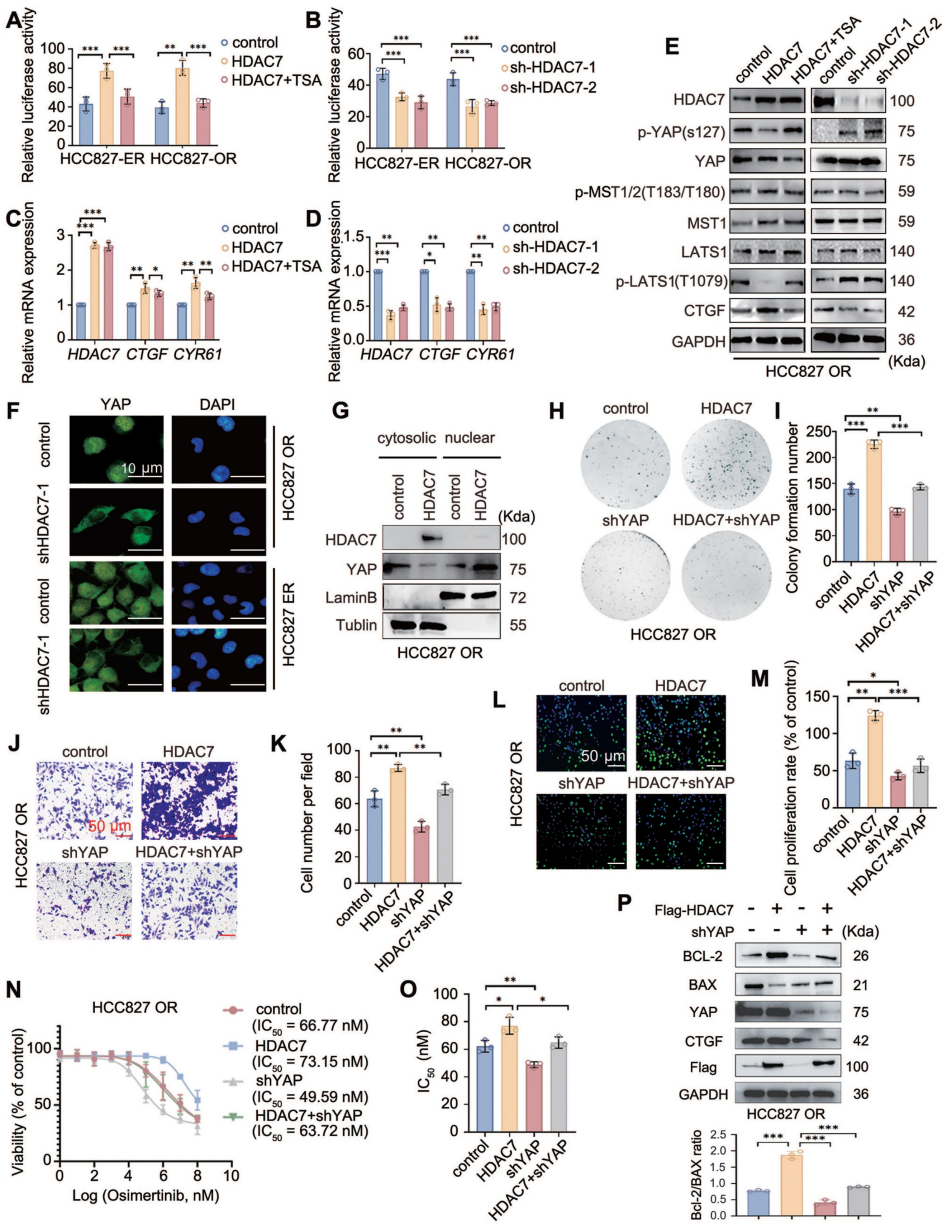
** HDAC7 promotes acquisition of a malignant phenotype and drug resistance by regulating the Hippo-YAP signalling pathway.** (A, B) Analysis of the activity of luciferase reporter genes in the Hippo signalling pathway. Overexpression of HDAC7 increases reporter gene activity, and this effect is reversed by TSA; knockdown of HDAC7 inhibits reporter gene activity. (C, D) RT‒qPCR detection of the mRNA expression of target genes downstream of the Hippo pathway. HDAC7 overexpression upregulated target gene expression, and TSA reversed this effect; moreover, HDAC7 knockdown inhibited transcription. (E) Western blot analysis of the levels of key proteins in the Hippo pathway (p-LATS1, LATS1, p-YAP, YAP, p-MST, MST, and CTGF) after the bidirectional regulation of HDAC7. (F, G) Immunofluorescence staining (F) and cytoplasmic separation (G) were performed to evaluate the level of nuclear YAP, and tubulin and LaminB were used as cytoplasmic and nuclear controls, respectively. (H, I) Colony formation assay. HDAC7 overexpression enhanced colony formation, and YAP knockdown reversed this effect. (J, K) Stromal gel invasion assay. HDAC7 overexpression increased cell invasion, and YAP knockdown reversed this effect (magnification: 200×). (L, M) Results of the EdU cell proliferation assay. HDAC7 overexpression increased cell proliferation, whereas YAP knockdown reversed this effect. (N, O) Analysis of the half maximal inhibitory concentration (IC_50_) of osimertinib. HDAC7 overexpression increased the IC_50_ value, and YAP knockdown decreased the IC_50_ value. (P) Western blot analysis of the levels of apoptosis-related proteins (cleaved caspase-3, cleaved PARP, Bcl-2, and Bax) after the overexpression of HDAC7 and knockdown of YAP. A histogram of the Bcl-2/Bax ratio was used to depict apoptosis. The data are presented as the means of three independent experiments. The columns represent the mean values, and the bars represent the standard deviations (SDs). *P < 0.05; **P < 0.01; and ***P < 0.001.

Whether TKI resistance is caused by the upregulation of HDAC7 through the inhibition of the activity of the Hippo pathway has not been reported in the literature. A luciferase reporter gene assay was performed to determine the possible effect of HDAC7 on Hippo signalling pathway activity, and the results revealed that HDAC7 overexpression increased the activity of TEAD, a core transcription factor of the Hippo signalling pathway, whereas TSA treatment abrogated this effect (**Figure 3A**). Conversely, HDAC7 knockdown had the opposite effect (**Figure 3B**). RT-qPCR revealed that overexpression of HDAC7 increased the expression of the mRNAs of the downstream target genes *CTGF* and *CYR61*, and the effects of HDAC7 overexpression on the activity of the Hippo pathway were reversed by TSA treatment and HDAC7 knockdown (**Figure 3C-D**). We subsequently assessed the effect of HDAC7 on the expression of core proteins in the Hippo signalling pathway by performing Western blot and found that the overexpression of HDAC7 significantly decreased the levels of the p-LATS1 (T1079) and p-YAP (Ser127) proteins, with the opposite results observed after the application of TSA or interference with HDAC7. In this process, neither the level of MST phosphorylation nor the level of total MST protein, LATS1, or total YAP were significantly altered (**Figure 3E, Figure S2A**). The nuclear translocation of YAP is a critical step in Hippo inhibition. Therefore, we assessed the effect of HDAC7 on the nucleoplasmic distribution of YAP by performing immunofluorescence staining and nucleoplasmic protein separation assays. The results revealed obvious intranuclear aggregation of YAP after the overexpression of HDAC7 (**Figure 3F-G, Figure S2B**). Taken together, these findings suggest that HDAC7 is a negative regulator of the Hippo pathway. We used shRNA-YAP and the YAP-TEAD inhibitor verteporfin in functional assays, colony formation assays (**Figure 3H-I, Figure S2C-D**), stromal gel invasion assays (**Figure 3J-K, Figure S2E-F**), EdU proliferation assays (**Figure 3L-M, Figure S2G-H**), and drug sensitivity testing (**Figure 3N-O, Figure S2I-J**) to further clarify the effect of HDAC7-mediated alterations in Hippo signalling on the malignant phenotype of tumour cells. Moreover, Western blot analysis revealed that the HDAC7 overexpression-induced increase in the Bcl-2/BAX ratio and CTGF expression was reversed by verteporfin or shRNA-YAP in the HCC827-ER cell line (**Figure 3P, Figure S2K**). Taken together, these results suggest that HDAC7 is a negative regulator of the Hippo signalling pathway and promotes the malignant phenotype and drug resistance of tumour cells by modulating the phosphorylation of LATS1 through the MST-independent pathway, thereby exerting a negative regulatory effect on the Hippo pathway.

### Identification of LATS1 kinase as an interacting protein of HDAC7

**Figure 4:**
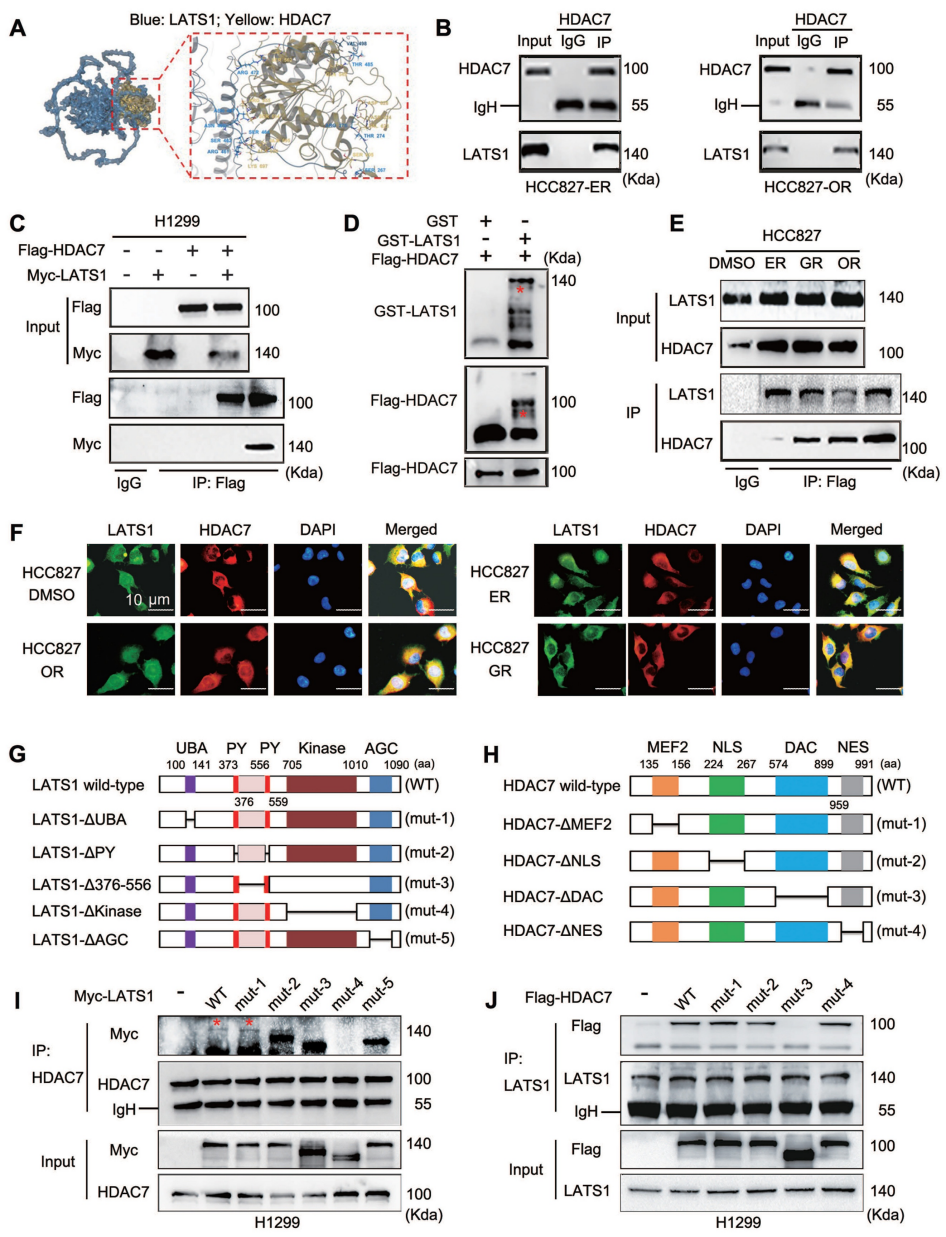
** Direct interaction of HDAC7 with LATS1 and identification of key binding structural domains.** (A) Molecular docking model of the HDAC7-LATS1 protein-protein interaction. (B) Endogenous HDAC7 interacts with LATS1 in HCC827-ER and HCC827-OR cells. HCC827-ER and HCC827-OR cell lysates were immunoprecipitated with anti-HDAC7 antibody or control IgG, and LATS1 expression was subsequently evaluated by anti-LATS1 immunoblotting. (C) Coimmunoprecipitation (Co-IP) analysis of exogenously expressed HDAC7 interacting with LATS1 in H1299 cells. (D) Purified GST-LATS1 was subjected to a GST pull-down assay, and its binding proteins were detected using Western blot. (E) Quantitative immunoprecipitation analysis was performed to compare the intensity of the HDAC7-LATS1 interaction in TKI-resistant cell lines with that in TKI-sensitive cell lines. (F) Immunofluorescence colocalization analysis showing the colocalization of HDAC7 and LATS1 in the cytoplasm. (G, H) Schematic representation of the structural domains of the LATS1 (G) and HDAC7 (H) proteins; the truncated mutant constructs used for functional studies are labelled. (I, J) Coimmunoprecipitation (Co-IP) analysis to identify key structural domains (LATS1-ΔKinase, HDAC7-ΔDAC) that mediate the HDAC7-LATS1 interaction (I: LATS1 mutant; J: HDAC7 mutant).

We transfected a Flag-HDAC7 plasmid into HCC827 cells, after which the protein complexes were collected by immunoprecipitation with a Flag antibody for subsequent mass spectrometry analysis to further explore the molecular mechanism through which HDAC7 affects the phosphorylation of LATS1 and inhibits Hippo pathway activity. We identified LATS1 as a binding protein of HDAC7. We verified the reliability of HDAC7 and LATS1 binding in HCC827-ER and HCC827-OR cells by performing molecular docking (**Figure 4A**) and endogenous immunoprecipitation experiments (**Figure 4B**). The results revealed that HDAC7 interacts with LATS1. In addition, we cotransfected HDAC7 and LATS1 plasmids into H1299 cells with high transfection efficiency, and this interaction was similarly confirmed by exogenous immunoprecipitation experiments (**Figure 4C**). *In vitro* recombinant protein-based GST-pulldown protein purification experiments revealed that purified GST-LATS1 could directly bind to HDAC7 (**Figure 4D**). We then compared the three resistant cell lines and observed that HDAC7 binds significantly more strongly to LATS1 in the three resistant cell lines than in the parental cell line HCC827 (**Figure 4E**). Immunofluorescence colocalization assays revealed that HDAC7 and LATS1 were colocalized in the cytoplasm in both parental HCC827 cells and resistant HCC827-GR/ER/OR lung cancer cells (**Figure 4F**). We further elucidated the structural basis of the interaction between HDAC7 and LATS1 by constructing corresponding spliceosomes based on pairs of known protein structural domains, including mutants of LATS1, such as Mutant-1, ΔUBA (ubiquitin binding domain, 100-141 aa deletion); Mutant-2, ΔPY × 2 (WW structural domain binding motif, 373-376 aa and 556-559 aa deletions); Mutant-3, ΔLinker (PY interconnecting region, 377-555 aa deletions); Mutant-4, ΔKinase (kinase catalytic domain, 705-1010 aa deletions); Mutant-5, ΔAGS (activation loop regulatory domain, 1011-1090 aa deletion) (**Figure 4G**); and mutants of HDAC7, such as Mutant-1, ΔMEF2 (135-156 aa deletion); Mutant-2, ΔNLS (nuclear localization signal, 224-267 aa deletion); Mutant-3, ΔDAC (deacetylase catalytic structural domain, 574-899 aa deletion); Mutant-4, ΔNES (nuclear export signal, 959-991 aa deletion)** (Figure 4H)**. By immunoprecipitation and immunoprotein blotting experiments, we first found that deletion of the kinase domain of LATS1 (LATS1-ΔKinase, Mutant-4) resulted in the loss of its ability to interact with HDAC7 (**Figure 4I**). On the other hand, when the deacetylase catalytic domain of HDAC7 was missing (HDAC7-ΔDAC, Mutant-3), it completely lost its ability to bind to LATS1 (**Figure 4J**). Therefore, these experimental results fully proved that the LATS1 protein binds to HDAC7 and revealed that the kinase domain of LATS1 binds to the DAC of HDAC7.

### HDAC7 promotes LATS1 deacetylation and thus decreases its phosphorylation

**Figure 5:**
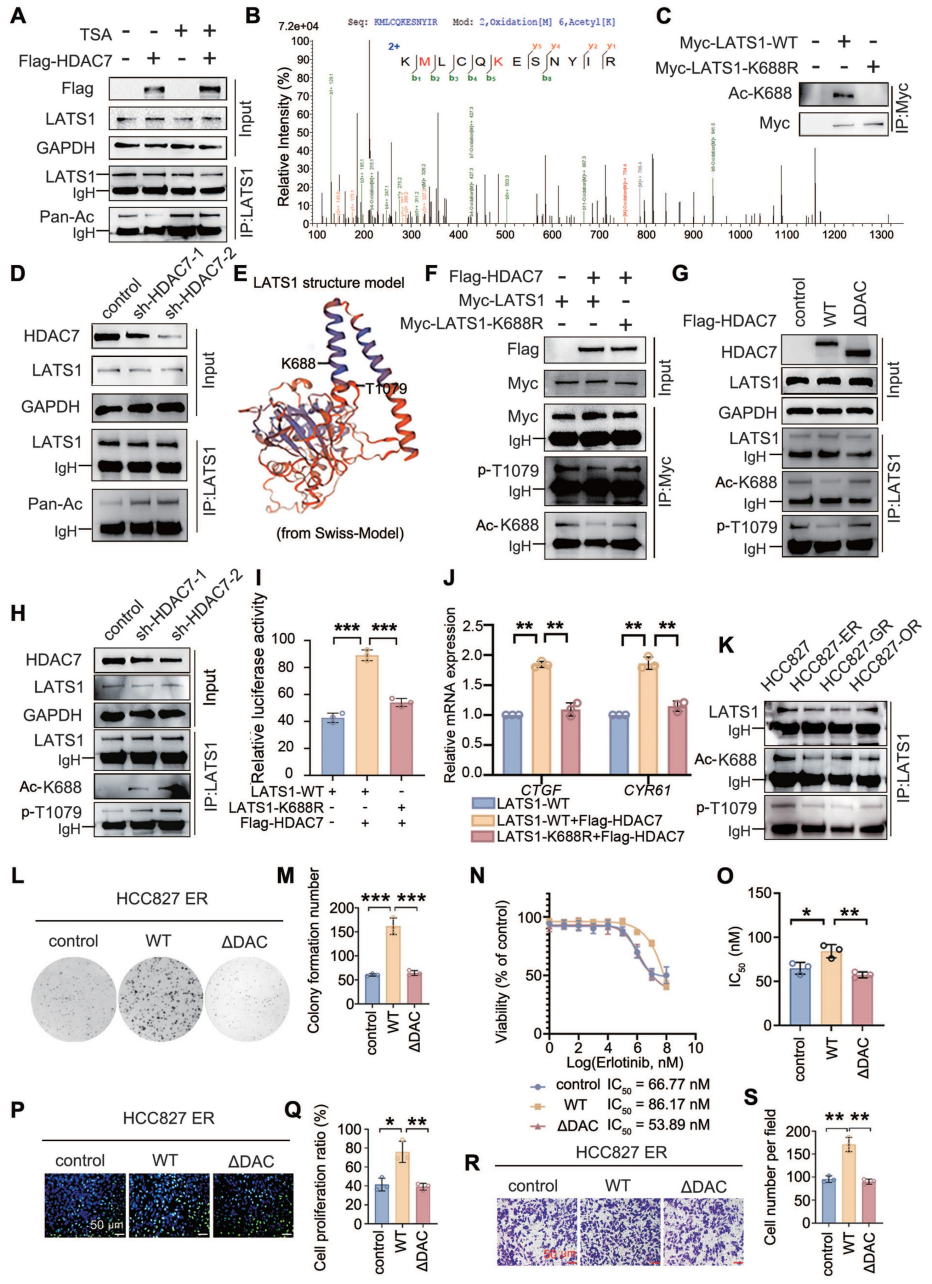
** HDAC7-mediated deacetylation of LATS1-K688 regulates its phosphorylation at T1079 and the cellular phenotype.** (A, D) HDAC7 was transfected into HCC827-OR cells, TSA was added, and the downregulation of the acetylation of LATS1 was detected by immunoprecipitation and immunoblotting; this effect was blocked by TSA. (B) Mass spectrometry analysis revealed LATS1-K688 as the site of HDAC7-mediated deacetylation. (C) Coimmunoprecipitation (Co-IP) verified the validity of the LATS1-K688 site-specific antibody. (E) Results from the SWISS-MODEL website showing that the LATS1-K688 acetylation site is spatially adjacent to the T1079 phosphorylation site. (F) Co-IP analysis revealed that the overexpression of HDAC7 reduced the level of K688 acetylation in LATS1-WT; this effect was abolished upon transfection of the LATS1-K688R mutant. (G) Co-IP analysis. HDAC7-WT overexpression reduced the level of LATS1 acetylation; this effect was abolished upon transfection of the HDAC7-ΔDAC (deacetylase activity deletion) mutant. (H) Knockdown of HDAC7 increased LATS1-K688 acetylation levels and decreased LATS1-T1079 phosphorylation levels. (I) Luciferase reporter assays revealed that HDAC7 overexpression suppressed YAP/TEAD transcriptional activity in cells expressing LATS1-WT, whereas this inhibitory effect was abolished in cells expressing the acetylation-mimetic LATS1-K688R mutant. (J) RT-qPCR analysis revealed that HDAC7 overexpression reduced the mRNA expression levels of the YAP target genes *CTGF* and *CYR61* in LATS1-WT-expressing cells but failed to do so in cells expressing the LATS1-K688R mutant. (K) Comparison of LATS1-K688 acetylation levels and LATS1-T1079 phosphorylation levels in TKI-resistant and TKI-sensitive cell lines. (L-M) Colony formation assay. HDAC7-WT overexpression increased colony formation, whereas the overexpression of HDAC7-ΔDAC abolished this effect. (N-O) Analysis of the IC_50_ of erlotinib. The overexpression of HDAC7-WT increased the IC_50_ value, whereas the overexpression of HDAC7-ΔDAC reduced the IC_50_ value. (P-Q) Results of the EdU cell proliferation assay. The overexpression of HDAC7-WT increased proliferation, whereas the overexpression of HDAC7-ΔDAC abolished this effect. (R-S) Stromal gel invasion assay. The overexpression of HDAC7-WT enhanced invasion, whereas the overexpression of HDAC7-ΔDAC abolished this effect. The data are presented as the means of three independent experiments. Columns indicate mean values, and bars indicate standard deviations (SDs). *P < 0.05; **P < 0.01; and ***P < 0.001.

On the basis of the intrinsic property of HDAC7 as a deacetylase and its ability to interact with LATS1, we further systematically investigated the regulation of LATS1 acetylation by HDAC7. The overexpression of HDAC7 in HCC827 cells significantly reduced the total acetylation level of LATS1, whereas TSA treatment counteracted the deacetylation of LATS1 by HDAC7 (**Figure 5A**). Conversely, compared with that of the control, the total acetylation level of LATS1 was significantly upregulated after HDAC7 interference (**Figure 5D**), indicating that LATS1 is a substrate for the activity of HDAC7. Accordingly, we further explored the specific acetylation site of LATS1 that is regulated by HDAC7. We transfected the HDAC7 plasmid into HCC827 cells and performed immunoprecipitation and mass spectrometry analyses; we detected the acetylation of lysine 688 of LATS1, which suggested that it is the site deacetylated by HDAC7 (**Figure 5B**). We commissioned Biologics to customize an antibody specific for the K688 site of LATS1 and validated the antibody using immunoprecipitation experiments (**Figure 5C**). Phosphorylation of threonine 1079 of LATS1 is required to activate the classical Hippo pathway. However, the effects of other posttranslational modifications of LATS1, especially acetylation, on its activity have rarely been reported. We found that lysine 688 in the LATS1 protein is adjacent to the threonine 1079 phosphorylation site in the three-dimensional spatial structure through the SWISS-MODEL website (**Figure 5E**). Therefore, we speculated that HDAC7 could influence the level of LATS1 phosphorylation by regulating its acetylation level and thus its phosphorylation level. HDAC7 was cotransfected with wild-type LATS1 and a plasmid carrying a LATS1 point mutation (lysine at position 688 was mutated to arginine, K688R) in HCC827 cells to explore this possibility, and immunoprecipitation revealed that HDAC7 downregulated the acetylation of K688 in wild-type LATS1, accompanied by a decrease in the phosphorylation of T1079, while these changes were abolished after transfection of the LATS1 mutant (K688R) (**Figure 5F**). Furthermore, we transfected wild-type HDAC7 and HDAC7-ΔDAC plasmids into HCC827 cells and found that compared with the empty vector, wild-type HDAC7 promoted LATS1-K688 deacetylation, which was accompanied by a decrease in its phosphorylation at the T1079 site; however, when cells were transfected with an HDAC7 mutant lacking the catalytic structural domain of the deacetylase (HDAC7-ΔDAC), the K688 acetylation level of LATS1 was restored to that of the control, and the T1079 phosphorylation level did not significantly change (**Figure 5G**). Similarly, the transfection of shRNA-HDAC7 resulted in the upregulation of LATS1-K688 acetylation and T1079 phosphorylation (**Figure 5H**). To further determine whether the acetylation status of K688 directly affects LATS1 activity, we conducted luciferase reporter gene experiments and RT‒qPCR to determine the effect of this substance on the activity of the Hippo signalling pathway. Luciferase reporter assays revealed that HDAC7 overexpression significantly suppressed YAP/TEAD transcriptional activity in cells expressing wild-type LATS1, whereas this inhibitory effect was abolished in cells expressing the LATS1-K688R mutant (**Figure 5I**). Consistently, RT-qPCR analysis demonstrated that HDAC7 overexpression reduced the mRNA expression levels of the YAP target genes *CTGF* and *CYR61* in LATS1-WT-expressing cells but did not exert these effects in cells expressing the LATS1-K688R mutant (**Figure 5J**), indicating that acetylation at K688 is required for HDAC7-mediated regulation of LATS1 activity.

In addition, in the collection of drug-resistant HCC827-ER/GR/OR cells, we observed that the level of LATS1-K688 acetylation in the drug-resistant cell lines was lower than the level of LATS1-T1079 phosphorylation in the nondrug-resistant HCC827 cell lines (**Figure 5K**). We transfected HDAC7-WT and HDAC7-ΔDAC into HCC827-ER cells to further determine the effect of the HDAC7-LATS1 interaction on the malignant phenotype of lung adenocarcinoma and drug sensitivity to TKIs, and the results of the colony formation assay, drug sensitivity assay, EdU proliferation assay, and stromal gel invasion assay revealed that overexpression of wild-type HDAC7 promoted colony formation in drug-resistant cells (**Figure 5L-M**), attenuated the sensitivity to gefitinib (increased IC_50_ value; **Figure 5N-O**), and proliferation (**Figure 5P-Q**) and increased the invasion (**Figure 5R-S**) of tumour cells. However, after the transfection of HDAC7-ΔDAC, which was unable to bind to LATS1 and lost the ability to deactivate LATS1 and because it lost its deacetylase activity, the aforementioned biological alterations disappeared. In summary, HDAC7 decreases the activity of the Hippo signalling pathway and enhances the malignant phenotype and drug resistance of lung adenocarcinoma in TKI-resistant cells by decreasing the acetylation of LATS1-K688 through binding to LATS1, which in turn suppresses LATS1 phosphorylation (T1079).

### EGFR nuclear translocation promotes the transcriptional activation of HDAC7 through the transcriptional coactivator role of the transcription factor STAT3

**Figure 6:**
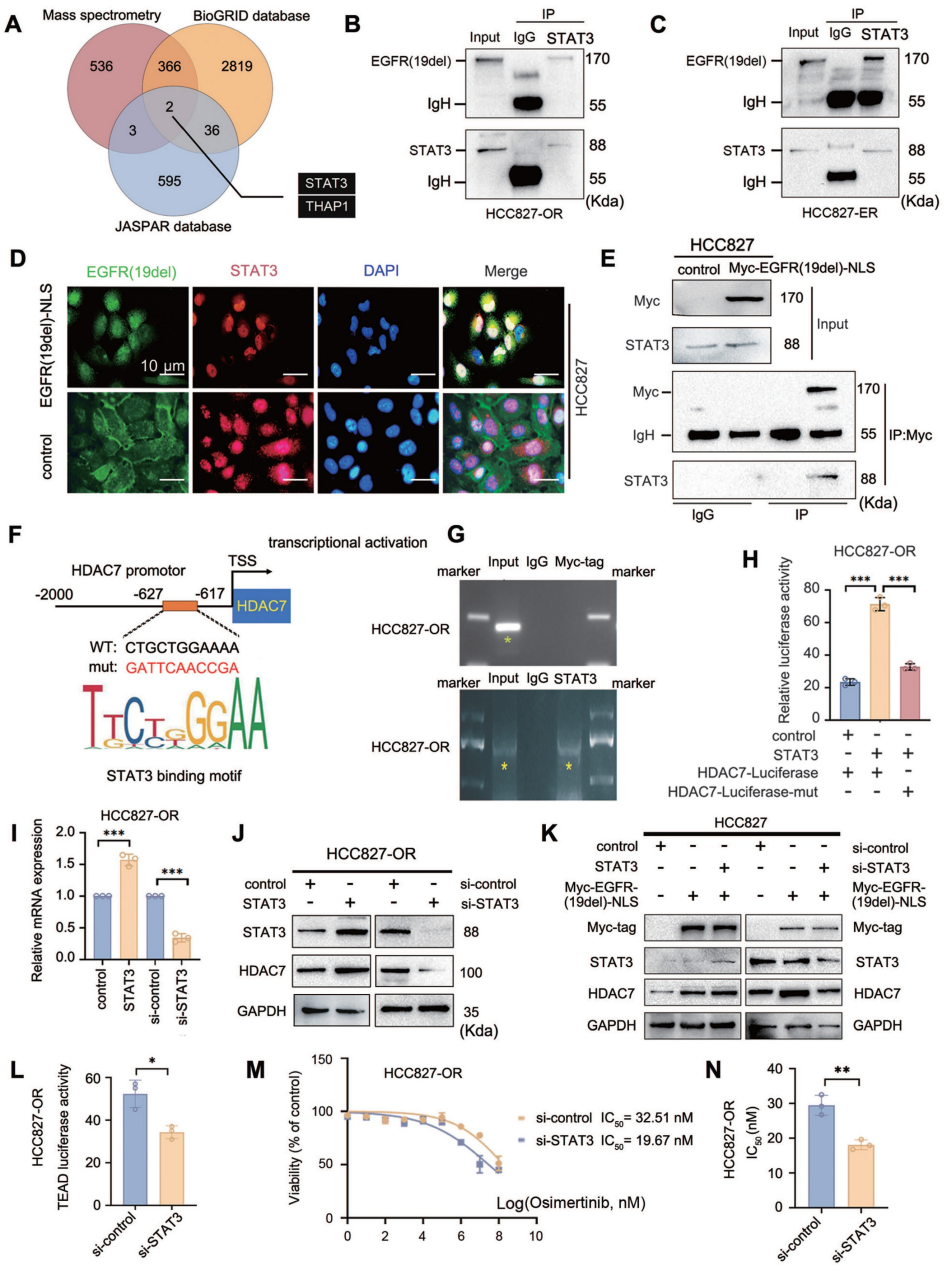
** EGFR nuclear translocation regulates STAT3-mediated HDAC7 transcription.** (A) An intersection analysis of the results from mass spectrometry and a database (BioGRID, JASPAR) revealed two transcription factors that potentially bind to EGFR. (B, C, E) Endogenous (B, C) and exogenous (E) immunoprecipitation analyses revealed that EGFR interacts with STAT3. (D) Immunofluorescence staining showing the colocalization of EGFR with STAT3 in the nucleus after transfection with EGFR(19del)-ΔNLS (nuclear localization deletion mutant). (F) Schematic representation of the HDAC7 promoter region. The STAT3 binding motif (CTGCTGGAAAA) is located between -627 and -617 bp relative to the transcriptional start site (TSS). (G) Chromatin immunoprecipitation (ChIP) assay demonstrating STAT3 binding to the HDAC7 promoter in HCC827-OR cells. Input, IgG, and STAT3 antibody pull-down samples were analysed via PCR. A Myc-tag antibody was used as a control. (H) Luciferase reporter gene assay. STAT3 overexpression significantly increased the activity of the wild-type HDAC7 promoter but did not significantly affect that of the mutant promoter. (I, J) RT‒qPCR (I) and Western blot (J) analyses. STAT3 overexpression upregulated HDAC7 mRNA and protein expression. (K) Western blot analysis showing that the knockdown of STAT3 eliminated the Myc-EGFR-19del-NLS-mediated upregulation of HDAC7 protein expression. (L) Luciferase reporter gene assay. Transfection of siSTAT3 significantly reduced the activity of the reporter gene. (M, N) Drug sensitivity analysis (IC_50_ determination). Knockdown of STAT3 reduced the half-maximal inhibitory concentration (IC_50_) of osimertinib. The data are presented as the means of three independent experiments. Columns indicate mean values, and bars indicate standard deviations (SDs). *P < 0.05; **P < 0.01; and ***P < 0.001.

Combined with our finding that HDAC7 expression was significantly upregulated in drug-resistant cells with EGFR translocation into the nucleus, we further explored the molecular mechanism through which the nuclear translocation of EGFR-19del causes HDAC7 overexpression. We hypothesize that the high expression of HDAC7 in TKI-resistant cells may also be due to the nuclear translocation of EGFR-19del, which synergizes with certain transcription factors to activate HDAC7 transcription. We screened the binding of the intranuclear mutant EGFR-19del protein to transcription factors using immunoprecipitation combined with mass spectrometry to verify this possibility. Since the current commercially available antibody against mutant EGFR-19del was not able to meet the requirements of the immunoprecipitation assay, we constructed a Myc-tagged EGFR-19del forced nuclear expression plasmid (Myc-EGFR-19del-NLS) to mimic the translocation of mutant EGFR-19del into the nucleus in drug-resistant cells. We subsequently collected protein complexes for detection by mass spectrometry using Myc-tagged monoclonal antibodies, and after nonspecifically bound proteins and proteins that were not explicitly annotated were removed, we obtained a set of 907 protein molecules that may bind to HDAC7. We searched BioGRID, a biologically universal interaction database, for 3223 transcription factors capable of binding to EGFR to further narrow its scope. Similarly, 636 proteins were identified in the Jaspar database of human-derived transcription factors. Taking the intersection of the results from the above three databases, we obtained the transcription factors signal transducer and activator of transcription 3 (STAT3) and THAP domain-containing protein 1 (THAP1) (**Figure 6A**). According to several reports in the literature, THAP1 is often associated with neurodevelopment and dystonia as a transcription factor 20-24, whereas STAT3 plays an important role as an oncogene in a variety of tumours and is closely associated with malignant progression and drug resistance in malignant tumours 25-28. Therefore, we chose STAT3 for our next study. The interaction of EGFR with STAT3 was demonstrated via endogenous immunoprecipitation experiments in HCC827-OR and HCC827-ER cells (**Figure 6B-C**). On the other hand, immunofluorescence staining revealed that after transfection of the Myc-EGFR-19del-NLS plasmid in HCC827 cells, the NLS sequence of mutant EGFR-19del was localized in the nucleus compared with the membrane localization of EGFR-19del in the empty vector-transfected group, and these results verified not only the validity of the plasmid but also the colocalization of EGFR-19del with STAT3 in the nucleus (**Figure 6D**). Moreover, exogenous immunoprecipitation verified the interaction between the two proteins (**Figure 6E**). All of the above results confirmed that EGFR-19del that translocated the nucleus can interact with the transcription factor STAT3. The JASPAR database suggests that STAT3 interacts with the HDAC7 promoter region from -627 bp to -617 bp (5'-CTGCTGGAAAA-3'), which had the greatest binding score (12.476). Accordingly, we designed corresponding ChIP primers and luciferase reporter gene plasmids (wild type/mutant) (**Figure 6F**). We first transfected the Myc-EGFR(del19)-NLS plasmid into HCC827-OR cells, and the ChIP results revealed that Myc-EGFR(del19)-NLS itself failed to enrich the sequence of the HDAC7 promoter region (including the -627/-617 bp region), while the ability of STAT3 to bind to the HDAC7 promoter region was verified using chromatin immunoprecipitation (**Figure 6G**).

Moreover, a luciferase reporter gene assay revealed that the transfection of STAT3 in HCC827-OR cells significantly increased the activity of the wild-type HDAC7 promoter reporter gene, whereas no significant change was detected in cells expressing the mutant promoter (**Figure 6H**); in contrast, STAT3 knockdown decreased the transcriptional activity of HDAC7 (**Figure S3A**). These results indicate that STAT3 can bind to the HDAC7 promoter region and increase its transcriptional activity. The results of RT-qPCR and Western blot assays revealed that the overexpression of STAT3 increased the mRNA and protein expression levels of HDAC7 (**Figure 6I-J**). We further verified the effect of the combination of both intranuclear mutant EGFR (19del) and STAT3 on the mRNA and protein expression levels of HDAC7 by performing Western blot, luciferase reporter gene and RT‒qPCR analyses after transfection of the Myc-EGFR(19del)-NLS sequence alone and cotransfection of Myc-EGFR(19del)-NLS and STAT3/si-STAT3 in HCC827 cells. Compared with those in the control group, the HDAC7 protein and mRNA levels and HDAC7 luciferase reporter gene activity in the Myc-EGFR(19del)-NLS alone group were significantly increased, and greater increases were observed in cells cotransfected with Myc-EGFR(19del)-NLS and STAT3 (**Figure 6K, Figure S3B, C**). Importantly, the Myc-EGFR (19del)-NLS-mediated promotion of HDAC7 expression and transcriptional activity disappeared when cells were transfected with si-STAT3 (**Figure 6L, Figure S3D, E**). Furthermore, dual-luciferase reporter assays demonstrated that STAT3 downregulation significantly reduced TEAD transcriptional activity (**Figure 6L**). Consistently, suppression of STAT3 decreased the IC₅₀ values of TKIs, including osimertinib, thereby restoring drug sensitivity in tumour cells (**Figure 6M-N**). Therefore, nuclear EGFR-19del acts as a “cotranscriptional activator” and cooperates with the transcription factor STAT3 to promote the transcriptional activity of HDAC7.

### *In vivo* experiments confirmed that HDAC7 knockdown or treatment with TSA can partially restore TKI sensitivity in drug-resistant cells

**Figure 7:**
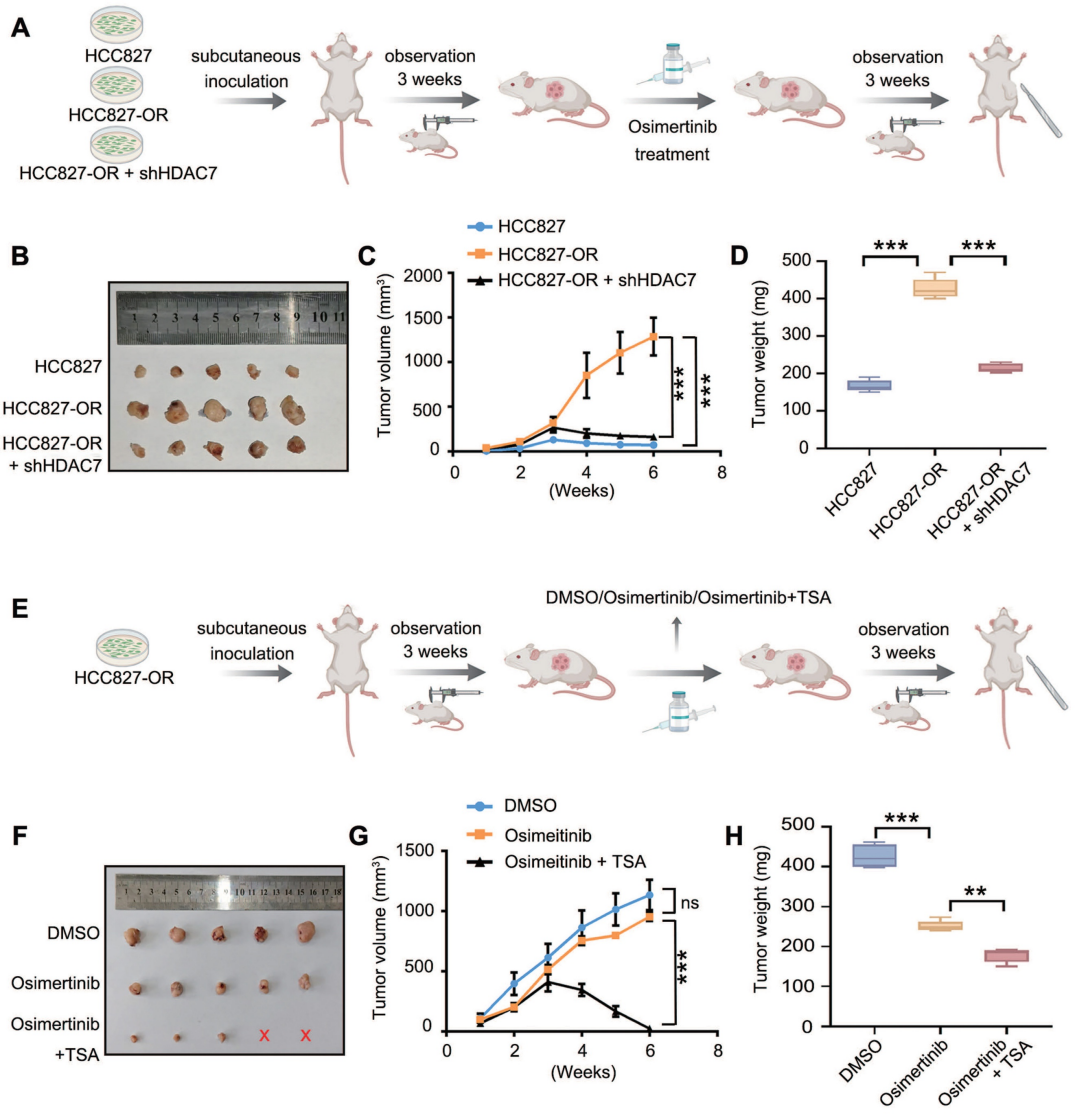
** Targeting HDAC7 restored the sensitivity of drug-resistant tumours to TKIs *in vivo*.** (A, E) Schematic diagrams of the design of tumour-bearing nude mice: (A) different cell inoculation groups (TKI-sensitive cells, osimertinib-resistant cells, and osimertinib-resistant cells with stable knockdown of HDAC7) and (E) different drug treatment groups (DMSO, osimertinib, and osimertinib combined with TSA). (B, F) Photographs of representative tumours from each group at the experimental endpoint. (C, G) Tumour growth curves. (C) Groups inoculated with different cells (HCC827, HCC827-OR, and shHDAC7-HCC827-OR cells) and treated with osimertinib (HCC827 cells + osimertinib vs. HCC827-OR cells + osimertinib vs. HCC827-OR cells + shHDAC7 + osimertinib). (G) After the HCC827-OR cells were inoculated, they were treated with DMSO, osimertinib, or osimertinib combined with TSA (*P* < 0.05, DMAO vs. osimertinib vs. osimertinib + TSA) (D, H) Tumour weights at the experimental endpoint. (D) Tumour weights were significantly lower in the HCC827-OR + shHDAC7 + osimertinib group than in the HCC827-OR + osimertinib group. (H) The tumour weight was significantly lower in the group treated with osimertinib combined with TSA than in the group treated with osimertinib alone. The data are presented as the means of three independent experiments. The columns represent the mean values, and the bars represent the standard deviations (SDs). *P < 0.05; **P < 0.01; and ***P < 0.001.

In the *in vivo* experiments, we subcutaneously injected HCC827 cells, HCC827-OR cells, and HCC827-OR + shHDAC7 cells into nude mice, which were allowed to grow for 3 weeks to promote tumour formation, after which they were treated with oxitinib administered to the oesophagus of each mouse. The growth of subcutaneous tumours in each group was observed for 3 weeks, the tumour growth curves were plotted, and the final weight was recorded (**Figure 7A**). The results revealed that the tumour volume and size in the HCC827-OR cell group were significantly greater than those in the HCC827 cell group, which indicated the greater proliferative ability and stronger TKI resistance of HCC827-OR cells. In contrast, the tumour volume and mass were significantly lower in the HCC827-OR cell group with HDAC7 knockdown than in the HCC827-OR group (**Figure 7B-D**), suggesting that the knockdown of HDAC7 could partially restore the sensitivity of drug-resistant cells to TKIs. Furthermore, we subcutaneously injected the HCC827-OR cell line into nude mice after treatment with DMSO (as a control), TKIs alone, or TKIs combined with TSA (**Figure 7E**) and found that the tumour mass and size in the TKI group did not change significantly compared with those in the DMSO group, whereas the combination of both TKIs and TSA resulted in significant reductions in tumour volume and weight (**Figure 7F-H**). Taken together, these *in vivo* experimental results suggest that HDAC7 plays an important role in promoting the malignant phenotype and drug sensitivity of EGFR-TKI-resistant cells and that the sensitivity of drug-resistant cells to TKIs can be partially restored by knocking down HDAC7 or inhibitor treatment, which can provide a corresponding experimental basis for the treatment of TKI resistance.

## Discussion

We previously elucidated and validated the molecular mechanism by which plasma-translocated EGFR inhibits Hippo activity 13. To date, whether nuclear EGFR can reverse the regulation of the Hippo pathway and further promote drug resistance remains unclear. In this study, the differences in expression between nonresistant and resistant cells were compared using high-throughput assays, and the results revealed that HDAC7 was generally highly expressed in different TKI-resistant cell lines. Multiple functional experiments conducted after the bidirectional regulation of HDAC7 revealed that HDAC7 has important biological functions in promoting the malignant phenotype of lung adenocarcinoma cells and TKI resistance. The literature shows that epigenetic changes in key factors in the Hippo signalling pathway cause the dysregulation of this pathway. For example, hypermethylation of the LATS2 promoter is prevalent in breast, lung, and other cancers, leading to its silencing and subsequent activation of YAP/TAZ, which promotes oncogenic signalling 29,30; moreover, histone modifications, including acetylation and methylation, further affect the expression and stability of Hippo pathway components. For example, histone deacetylase (HDAC) deacetylates YAP, which stabilizes it and promotes its nuclear translocation, thereby driving the transcription of genes associated with cell proliferation and survival 31. The effect of LATS1 acetylation on its activity has not yet been reported. In the present study, we observed and verified that HDAC7 can bind to LATS1 and promote deacetylation at the LATS1-K688 site, which further decreases the phosphorylation of the LATS1-T1079 site and negatively regulates Hippo pathway activity, thus exacerbating resistance to TKIs. These findings provide new insights into the molecular mechanisms underlying the effects of HDAC7 on TKI resistance. Although we confirmed that K688 is the key deacetylation site through which HDAC7 regulates LATS1 function, we cannot completely exclude the contribution of other potential acetylation sites, such as K683, which was predicted by PhosphoSitePlus. Furthermore, absolute quantification analysis of the acetylated peptides at the K688 site using quantitative mass spectrometry will further validate our conclusions. These aspects remain to be elucidated in the future through more systematic site mutation screening and dynamic modification proteomics analysis.

Notably, the molecular signalling pathway described in our study differs significantly from the previously reported EGFR-SIK2-MST-LATS-YAP molecular transduction signalling pathway 13, which focuses primarily on the regulatory role of EGFR-19del translocation into the cytoplasm. The results of the current study reveal mainly the molecular regulatory mechanism through which EGFR-19del in the nucleus exacerbates the resistance of lung adenocarcinoma to TKIs. Hence, EGFR-19del, located in different subcellular compartments, consequently promotes the activation of YAP through these two pathways, which can have independent or and interconnected effects, thereby promoting malignant progression and TKI resistance in lung adenocarcinoma with EGFR mutations.

On the other hand, we further explored the reason for the high expression of HDAC7 in TKI-resistant cells. Previous studies have shown that the activation of EGFR ultimately leads to its translocation into the nucleus 14, suggesting that EGFR can exist in various locations in the cell membrane, cytoplasm and nucleus. These findings also imply that the different subcellular locations of EGFR may result in differences in sensitivity to TKI analogues. In view of this phenomenon, we identified the binding of the transcription factor STAT3 to EGFR-19del through mass spectrometry analysis and a protein interaction database and verified that nuclear EGFR-19del can act as a “transcriptional coactivator” to promote the transcription of HDAC7 in cooperation with STAT3, thus answering the question of how HDAC7 is upregulated in TKI-resistant cells. Importantly, endogenous and exogenous co-immunoprecipitation assays, together with immunofluorescence analysis, demonstrated that EGFR-19del interacts with STAT3 and colocalizes with STAT3 in the nucleus following Myc-EGFR(del19)-NLS expression. ChIP assays further revealed that STAT3 directly binds to the -627/-617 bp region of the HDAC7 promoter and significantly enhances its transcriptional activity. In contrast, ChIP analysis of Myc-EGFR(del19)-NLS showed no enrichment at the HDAC7 promoter region, including the STAT3-binding site. These findings indicate that EGFR-19del does not directly bind to the HDAC7 promoter. Rather, EGFR functions as a nuclear transcriptional coactivator that cooperates with STAT3 to enhance STAT3-mediated transcriptional activation of HDAC7, instead of acting as a DNA-binding transcription factor. Notably, the reasons for the upregulation of HDAC7 expression in tumour cells are multifaceted, and there is still much room for exploring whether EGFR-19del that has translocated into the plasma or nucleus can regulate the expression of HDAC7 through other molecular mechanisms remains.

Studies on the mechanism of drug resistance after TKI treatment ultimately aim to prevent or reverse resistance, and recent studies have shown that the aberrant expression of HDAC is associated with the emergence of drug resistance after TKI treatment and that the use of HDAC inhibitors (e.g., SAHA) can also reverse resistance to a certain extent. The underlying mechanism is related to apoptosis, but other mechanistic details are largely unknown 32. We confirmed the mechanism by which mutant EGFR enters the cell nucleus after TKI-induced resistance and induces high expression of HDAC7, as well as the mechanism by which HDAC7 inhibits the activity of the Hippo pathway through LATS1 and causes resistance, and proposed a new strategy for targeted application of HDAC inhibitors to reverse TKI resistance.

## Conclusions

This study reveals a cascade mechanism—the nuclear entry of EGFR-19del drives drug resistance in lung adenocarcinoma through the inhibition of Hippo signalling via the STAT3-HDAC7-LATS1 signalling axis. These findings provide experimental evidence for the combined application of TKIs and HDAC inhibitors to prevent or reverse the emergence of TKI resistance during lung adenocarcinoma treatment.

## Supplementary Material

Supplementary methods, figures and tables.

## Figures and Tables

**Table 1:** The sequences of the primers used for RT‒qPCR

Primer sequences (5′→3′)	
*HDAC7*	Forward 5′- GGCGGCCCTAGAAAGAACAG -3′ Reverse 5′- CTTGGGCTTATAGCGCAGCTT -3′
*CTGF*	Forward 5′- CTTGCGAAGCTGACCTGGAAGA -3′ Reverse 5′- CCGTCGGTACATACTCCACAGA -3′
*CYR61*	Forward 5′- GGAAAAGGCAGCTCACTGAAGC -3′ Reverse 5′- GGAGATACCAGTTCCACAGGTC -3′
*GAPDH*	Forward 5′- GTCTCCTCTGACTTCAACAGCG -3′ Reverse 5′- ACCACCCTGTTGCTGTAGCCAA -3′

**Table 2:** Association between the subcellular location of mutant EGFR and HDAC7 in adenocarcinoma with EGFR gene mutation (19del)

		EGFR-19del subcellular location			Pearson's Chi-Square	P value
		cytosolic/nuclear	membrane	Total		(Fisher)
HDAC7	Negative	2	17	19		
	Positive	16	25	41	5.021	0.034*
	Total	18	42	60		

*: statistically significant

## Data Availability

Data supporting the results of this study may be obtained from the corresponding author upon reasonable request.

## Abbreviations

AGS: activation loop regulatory domain
AMOT: angiomotin family proteins
BAX: bcl-2-Associated X protein
Bcl-2: B-cell lymphoma 2
BCRP: breast cancer resistance protein
CCNE: Cyclin E
CM: choroidal melanoma
c-MYC: cellular myelocytomatosis oncogene
CTGF: connective tissue growth factor
Cyr61: cysteine-rich angiogenic inducer 61
FATs: FAT atypical cadherins
DAC: deacetylation
EDTA: ethylenediaminetetraacetic acid
EGFR: epidermal growth factor receptor
HDAC7: histone deacetylase 7
LATS1: large tumour suppressor kinase 1
MEF2: myocyte enhancer factor 2
MOB1: MOB kinase activator 1
MST: mammalian ste20-like kinase
NF2: neurofibromin 2
NLS: nuclear localization signal
NES: nuclear export signal
PARP: poly (ADP-ribose) polymerase
PBS: phosphate-buffered saline
PFS: progression-free survival
PMSF: phenylmethanesulfonyl fluoride
PY: WW structural domain binding motif
RT‒qPCR: reverse transcription quantitative polymerase chain reaction
SAV1: salvador family
SD: standard deviation
SIK2: salt-inducible kinase 2
STAT3: signal transducer and activator of transcription 3
STR: short tandem repeat
TSA: trichostatin A
TEAD: transcriptional enhanced associate domain
TAZ: transcriptional coactivator with PDZ-binding motif
THAP1: THAP domain-containing protein 1
TKI: tyrosine kinase inhibitor
UBA: ubiquitin binding domain
WWCs: WWC family proteins
YAP: yes-associated protein
