# Supplementary material for: EGFR-19del nuclear translocation increases HDAC7 expression inhibiting the Hippo pathway and exacerbating TKI resistance in lung adenocarcinoma
Source: Theranostics. 2026 Apr 8;16(11):5992–6010. doi: 10.7150/thno.128873 (PMC13142111; doi:10.7150/thno.128873)
Supplement: Supplementary file 1 — Supplementary methods, figures and tables. [file thnov16p5992s1.pdf]

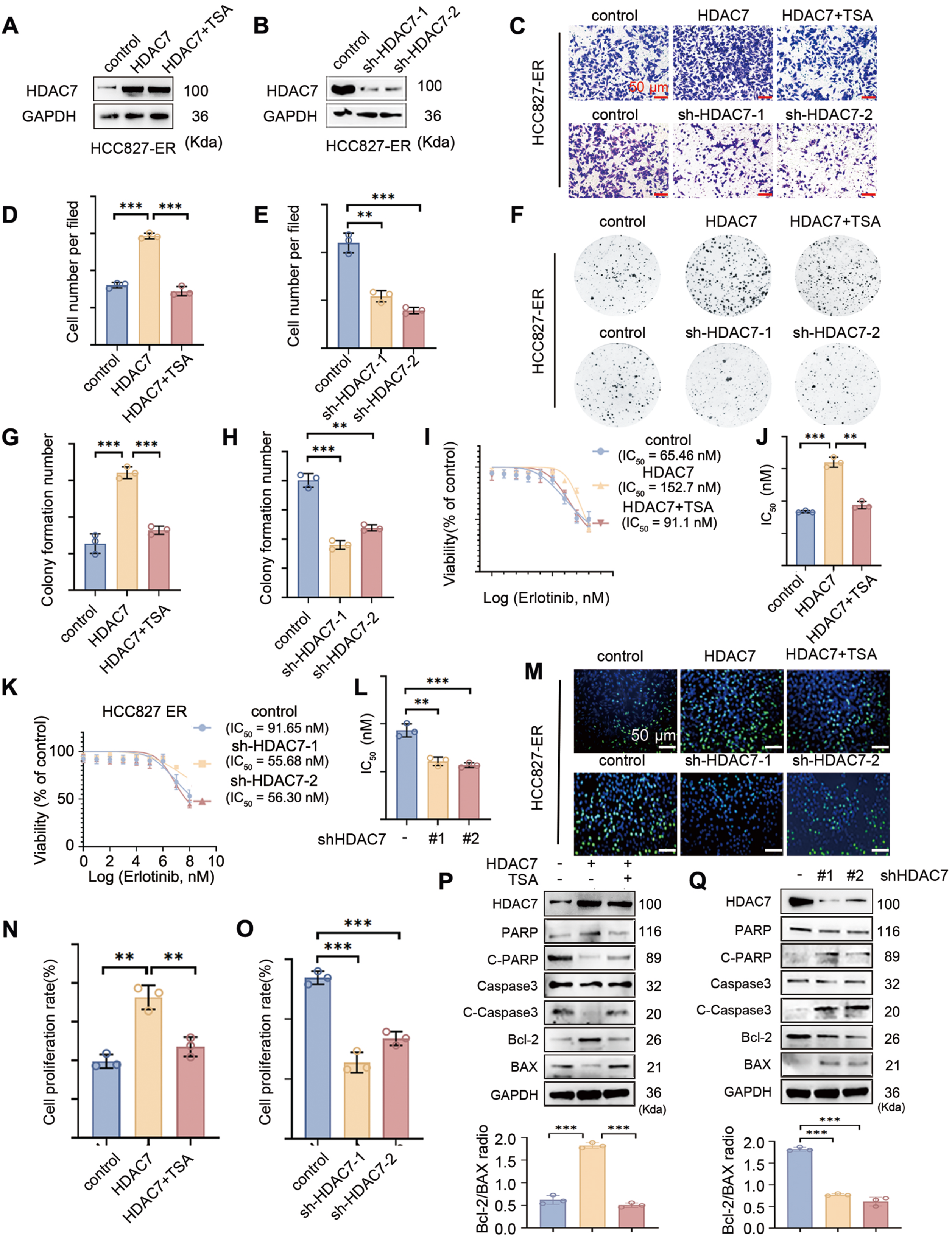
A
control
HDAC7
HDAC7+TSA
HDAC7
GAPDH
100
36
HCC827-ER
(Kda)
B
control
sh-HDAC7-1
sh-HDAC7-2
HDAC7
GAPDH
100
36
HCC827-ER
(Kda)
C
control
HDAC7
HDAC7+TSA
control
sh-HDAC7-1
sh-HDAC7-2
HCC827-ER
50 μm
D
Cell number per filed
***
control
HDAC7
HDAC7+TSA
E
Cell number per filed
**
***
control
sh-HDAC7-1
sh-HDAC7-2
F
control
HDAC7
HDAC7+TSA
control
sh-HDAC7-1
sh-HDAC7-2
HCC827-ER
G
Colony formation number
***
control
HDAC7
HDAC7+TSA
H
Colony formation number
**
***
control
sh-HDAC7-1
sh-HDAC7-2
I
Viability(% of control)
Log (Erlotinib, nM)
control (IC50 = 65.46 nM)
HDAC7 (IC50 = 152.7 nM)
HDAC7+TSA (IC50 = 91.1 nM)
J
IC50 (nM)
***
**
control
HDAC7
HDAC7+TSA
K
HCC827 ER
Viability (% of control)
Log (Erlotinib, nM)
control (IC50 = 91.65 nM)
sh-HDAC7-1 (IC50 = 55.68 nM)
sh-HDAC7-2 (IC50 = 56.30 nM)
L
IC50 (nM)
**
***
shHDAC7
-
#1
#2
M
control
HDAC7
HDAC7+TSA
control
sh-HDAC7-1
sh-HDAC7-2
HCC827-ER
50 μm
N
Cell proliferation rate(%)
**
control
HDAC7
HDAC7+TSA
O
Cell proliferation rate(%)
***
control
sh-HDAC7-1
sh-HDAC7-2
P
HDAC7
TSA
HDAC7
PARP
C-PARP
Caspase3
C-Caspase3
Bcl-2
BAX
GAPDH
100
116
89
32
20
26
21
36
(Kda)
Bcl-2/BAX radio
***
Q
-
#1
#2
shHDAC7
HDAC7
PARP
C-PARP
Caspase3
C-Caspase3
Bcl-2
BAX
GAPDH
100
116
89
32
20
26
21
36
(Kda)
Bcl-2/BAX radio
***

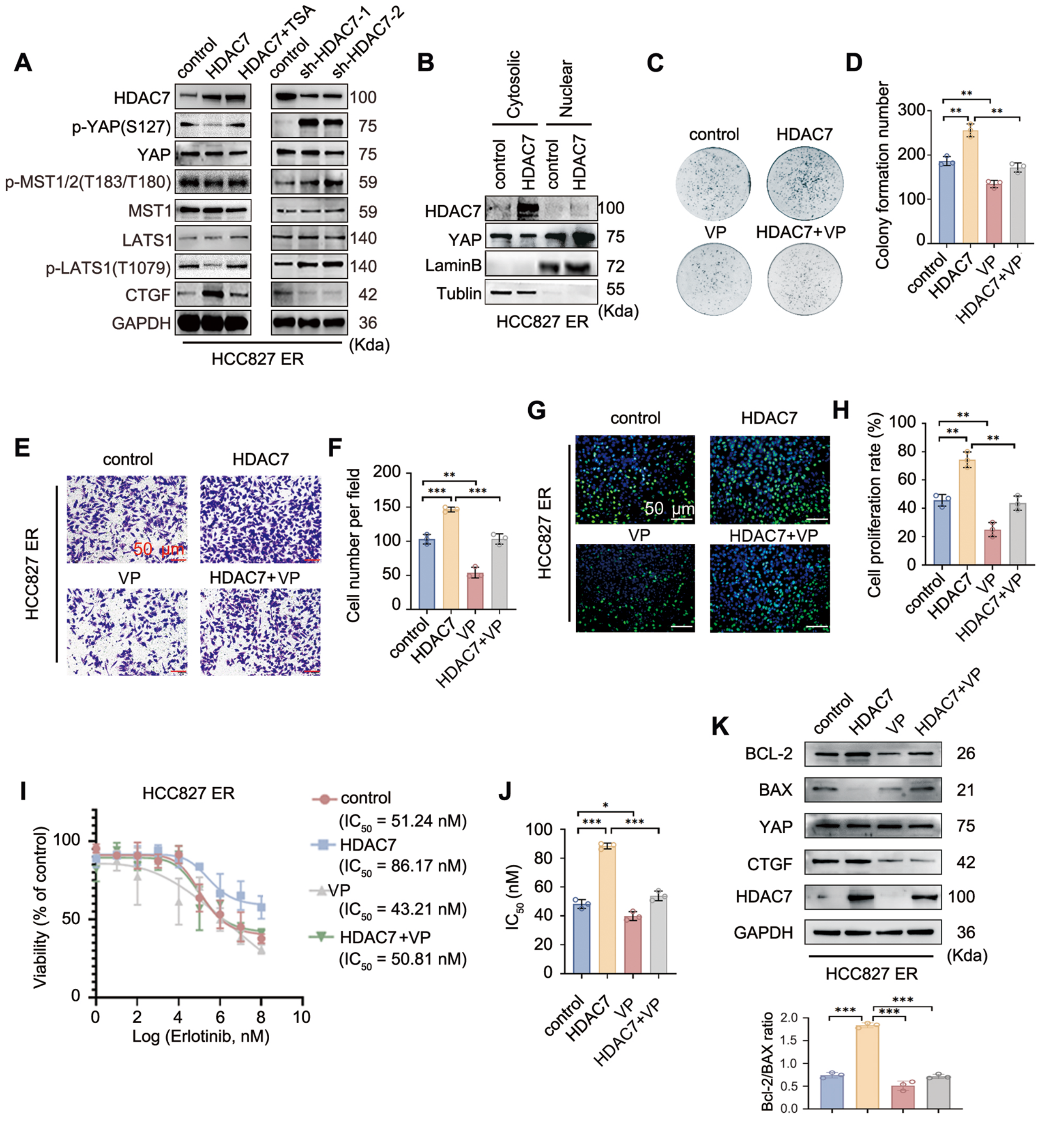
A
control
HDAC7
HDAC7+TSA
control
sh-HDAC7-1
sh-HDAC7-2
HDAC7
p-YAP(S127)
YAP
p-MST1/2(T183/T180)
MST1
LATS1
p-LATS1(T1079)
CTGF
GAPDH
100
75
75
59
59
140
140
42
36
(Kda)
HCC827 ER
B
Cytosolic
Nuclear
control
HDAC7
control
HDAC7
HDAC7
YAP
LaminB
Tublin
100
75
72
55
(Kda)
HCC827 ER
C
control
HDAC7
VP
HDAC7+VP
D
Colony formation number
control
HDAC7
VP
HDAC7+VP
E
control
HDAC7
VP
HDAC7+VP
HCC827 ER
50 μm
F
Cell number per field
control
HDAC7
VP
HDAC7+VP
G
control
HDAC7
VP
HDAC7+VP
HCC827 ER
50 μm
H
Cell proliferation rate (%)
control
HDAC7
VP
HDAC7+VP
I
HCC827 ER
Viability (% of control)
Log (Erlotinib, nM)
control (IC50 = 51.24 nM)
HDAC7 (IC50 = 86.17 nM)
VP (IC50 = 43.21 nM)
HDAC7+VP (IC50 = 50.81 nM)
J
IC50 (nM)
control
HDAC7
VP
HDAC7+VP
K
control
HDAC7
VP
HDAC7+VP
BCL-2
BAX
YAP
CTGF
HDAC7
GAPDH
26
21
75
42
100
36
(Kda)
HCC827 ER
Bcl-2/BAX ratio

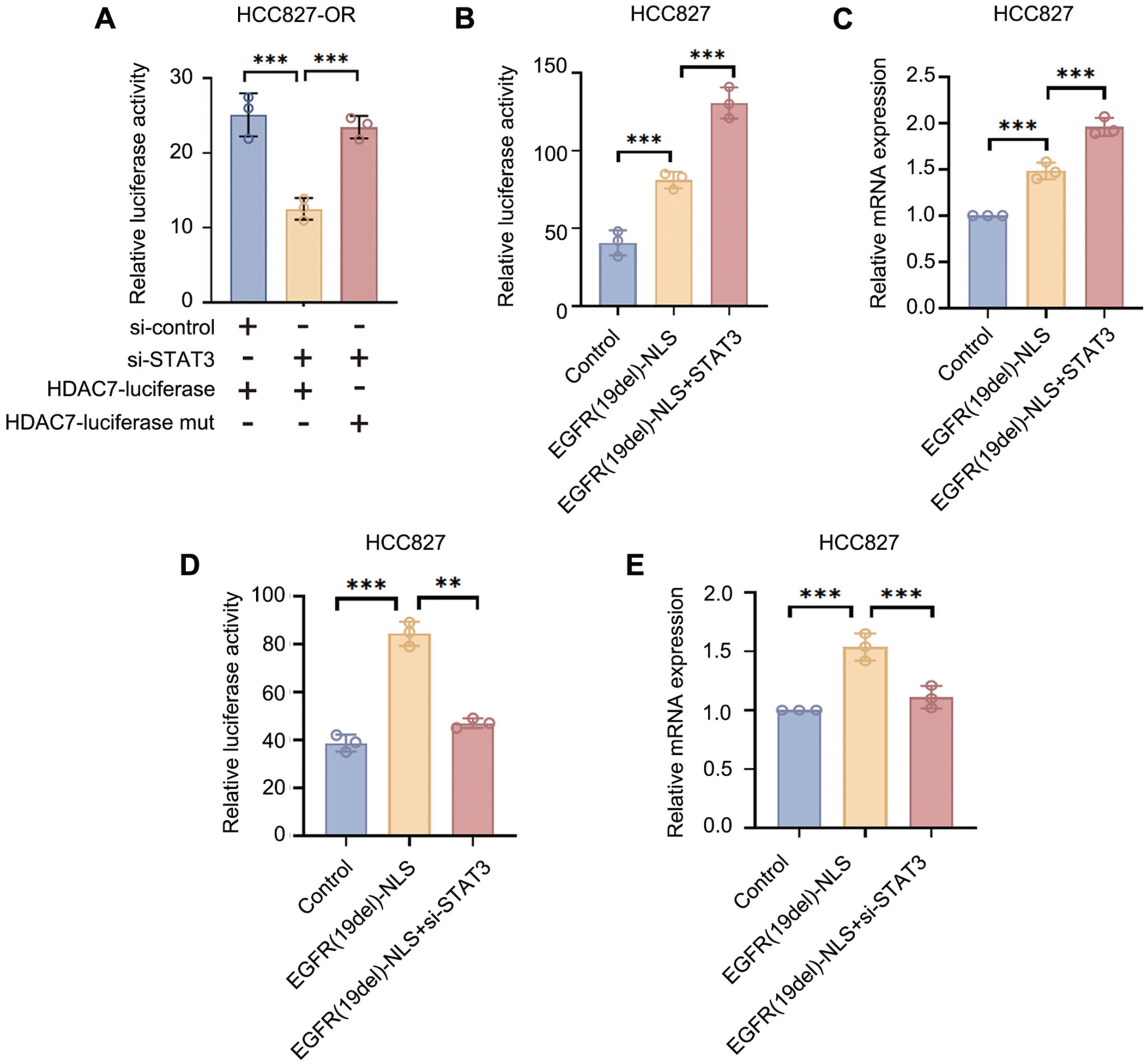
A
HCC827-OR
Relative luciferase activity
30
20
10
0
***
***
si-control + - -
si-STAT3 - + +
HDAC7-luciferase + + -
HDAC7-luciferase mut - - +
B
HCC827
Relative luciferase activity
150
100
50
0
***
***
Control
EGFR(19del)-NLS
EGFR(19del)-NLS+STAT3
C
HCC827
Relative mRNA expression
2.5
2.0
1.5
1.0
0.5
0.0
***
***
Control
EGFR(19del)-NLS
EGFR(19del)-NLS+STAT3
D
HCC827
Relative luciferase activity
100
80
60
40
20
0
***
**
Control
EGFR(19del)-NLS
EGFR(19del)-NLS+si-STAT3
E
HCC827
Relative mRNA expression
2.0
1.5
1.0
0.5
0.0
***
***
Control
EGFR(19del)-NLS
EGFR(19del)-NLS+si-STAT3

Supplementary Figure Captions

Supplementary Figure S1: HDAC7 promotes a malignant phenotype and reduces the sensitivity of HCC827-ER cells to TKIs. (A, B) Western blotting analysis of HDAC7 overexpression and knockdown efficiency in HCC827-ER cells. (C-E) Stromal gel invasion assay. HDAC7 overexpression enhanced cell invasion, and the HDAC inhibitor tretinoin (TSA) reversed this effect; HDAC7 knockdown inhibited invasion (magnification: 200×). (F-H) Cell colony formation assay. HDAC7 overexpression enhanced colony formation, and TSA reversed this effect; HDAC7 knockdown inhibited colony formation. (I-L) Drug sensitivity analysis (IC_{50} assay). HDAC7 overexpression increased the half maximal inhibitory concentration (IC_{50}) of erlotinib, and TSA reversed this effect; HDAC7 knockdown decreased the IC_{50}. (M-O) Results of the EdU cell proliferation assay. HDAC7 overexpression increased the proliferation of the cells, and TSA reversed this effect; HDAC7 knockdown reduced proliferation. (P, Q) Bidirectional regulation of cell proliferation. Western blotting analysis of the bidirectional regulation of apoptosis-related protein (cleaved caspase-3, cleaved PARP, Bcl-2, and Bax) levels after the modulation of HDAC7 expression. Histogram of Bcl-2/Bax was used to detect the apoptosis of cells. The data are presented as the means of three independent experiments. The columns represent the mean values, and the bars represent the standard deviations (SDs). $^*P < 0.05$; $^{**}P < 0.01$; and $^{***}P < 0.001$.

Supplementary Figure S2: HDAC7 promotes malignant phenotype and drug resistance in HCC827-ER cells by regulating Hippo pathway. (A) Western blotting analysis of the levels of key proteins in the Hippo pathway (p-LATS1, LATS1, p-YAP, YAP, p-MST, MST, and CTGF) after the bidirectional regulation of HDAC7. (B) Nucleoplasmic separation assay to detect the subcellular distribution of YAP after the overexpression of HDAC7. (C, D) Colony formation assay. HDAC7 overexpression enhanced colony formation, and YAP knockdown reversed this change. (E, F) Stromal gel invasion assay. HDAC7 overexpression increased cell invasion, and YAP knockdown reversed this effect (magnification: 200×). (G, H) Results of the EdU cell proliferation assay. HDAC7 overexpression increased cell proliferation, whereas YAP knockdown reversed this effect. (I, J) Analysis of the half maximal inhibitory concentration (IC_{50}) of erlotinib. HDAC7 overexpression increased the IC_{50} value, and YAP knockdown reduced the IC_{50}. (K) Western blotting analysis of the levels of apoptosis-related proteins (cleaved caspase-3, cleaved PARP, Bcl-2, and Bax) after the overexpression of HDAC7 and knockdown of YAP. Histogram of Bcl-2/Bax was used to detect the apoptosis of cells. The data are presented as the means of three independent experiments. The columns represent the mean values, and the bars represent the standard deviations (SD)s. *$P < 0.05$; **$P < 0.01$; and ***$P < 0.001$.

Supplementary Figure S3: Role of STAT3 in the EGFR(19del)-NLS-mediated transcriptional activation of HDAC7. (A) Luciferase reporter gene analysis. STAT3 knockdown decreased the activity of the wild-type HDAC7 promoter but did not

significantly affect the activity of the mutant promoter. (B, C) Luciferase reporter gene (B) and RT–qPCR (C) analyses. The transfection of Myc-EGFR(19del)-NLS increased HDAC7 promoter activity and mRNA expression; cotransfection of Myc-EGFR(19del)-NLS with STAT3 further increased their levels. (D, E) Luciferase reporter gene (D) and RT–qPCR (E) analyses. STAT3 knockdown eliminated the Myc-EGFR(19del)-NLS-induced upregulation of HDAC7 promoter activity and mRNA expression. The data are presented as the means of three independent experiments. Columns indicate mean values, and bars indicate standard deviations (SDs). $^{*}P < 0.05$; $^{**}P < 0.01$; and $^{***}P < 0.001$.

Supplementary Materials

Plasmids, Short-hairpin RNA (shRNA), Small interfering RNA (shRNA) and Reagents

The plasmids, shRNA and siRNA information involved in this study is listed as follows: shRNA HDAC7 (no.51564); siRNA STAT3 (no.6774) was purchased from OriGene Company (Wuxi, China). pCMV-NLS-EGFR (human)-NLS-3×Myc-Neo (no.G57050); pCMV-LATS1 (human)-3×Myc-Neo; pCMV-LATS1 (human)-3×Myc-Neo (no.P68214); pCDNA3.1-3×Myc-Neo (no.P14395); pCMV-LATS1 (human)-del(100-141aa)- 3×Myc-Neo (no.G47483); pCMV-LATS1 (human)-del(373-376aa) & (556-559aa)- 3×Myc-Neo (no.G47484); pCMV-LATS1 (human)-del(705-1010aa)-3×Myc-Neo (no.G47485); pCMV-LATS1 (human)-del(376-556aa)-3×Myc-Neo (no.G47492); pLV3-CMV-HDAC7 (human)-FLAG-CopGFP-Puro (no.P73827); pCDH-CMV-MCS-FLAG-EF1-CopGFP-T2A-Puro (no.P63649); pCMV-HDAC7 (human)-del(135-156aa)-3×FLAG-Neo (no.G47487); pCMV-HDAC7 (human)-del(224-267aa)-3×FLAG-Neo (no.G47488); pCMV-HDAC7 (human)-del(574-899aa)-3×FLAG-Neo (no.G47489); pCMV-HDAC7 (human)-del(959-991aa)-3×FLAG-Neo (no.G47490) were purchased from MiaoLing Biotechnology Co., Ltd (China). Verteporfin (CL318952) and Trichostatin A (TSA, HY-15144) were purchased from MedChemExpress company (Shanghai, China).

Antibodies

The antibodies includes: HDAC7 (1:500, #33418, Cell Signaling

Technology/CST); HDAC7 (1:500, 26207-1-AP, Proteintech); MST (1:500, #14946, CST); p-MST (1:500, #49332, CST); LATS1 (1:500, #3477, CST); p-LATS1 (1:500, #8654, CST); YAP (1:500, #14074, CST); p-YAP (1:500, #13008, CST); CTGF (1:200, sc-365970, Santa Cruz Technology); GAPDH (1:1,000; 60004-1-Ig; Proteintech); STAT3 (1:500, #9139, CST); Myc-tag (1:1000, 2276, CST); GST-tag (1:1000, 2622, CST); Flag-tag (1:1000, 14793, CST); EGFR-19del (1:500, #2085, CST); BCL-2 (1:1000, 26593-1-AP, Proteintech); BAX(1:1000, 50599-2-Ig, Proteintech); Caspase3 (1:1000, 66470-2-Ig, Proteintech); cleaved-Caspase3 (1:500, 25128-1-AP, Proteintech); PARP (1:1000, 13371-1-AP, Proteintech); cleaved-PARP (1:1000, 60555-1-Ig, Proteintech); Acetylated-Lysine Antibody (1:500, #9441, CST); LATS1- lysine 688 was prepared by Bioss Antibodies Company (Beijing, China).

Supplementary Table S1. The sequences of primers used for RT-qPCR

Primer sequences (5′→3′)	
HDAC7	Forward 5′- GGCGGCCCTAGAAAGAACAG -3′
	Reverse 5′- CTTGGGCTTATAGCGCAGCTT -3′
CTGF	Forward 5′- CTTGCGAAGCTGACCTGGAAGA -3′
	Reverse 5′- CCGTCGGTACATACTCCACAGA -3′
CYR61	Forward 5′- GGAAAAGGCAGCTCACTGAAGC -3′
	Reverse 5′- GGAGATACCAGTTCCACAGGTC -3′
GAPDH	Forward 5′- GTCTCCTCTGACTTCAACAGCG -3□
	Reverse 5′- ACCACCCTGTTGCTGTAGCCAA -3

Supplementary Table S2: The association between mutant EGFR subcellular location and HDAC7 in adenocarcinoma with EGFR mutation (19del)

		EGFR-19del subcellular location			Pearson's Chi-Square	*P*-value
		cytosolic/nuclear	membrane	Total		(Fisher)
HDAC7	Negative	2	17	19		
	Positive	16	25	41	5.021	0.034*
	Total	18	42	60		

*: statistically significant
